# Isoflavones, anthocyanins, phenolic content, and antioxidant activities of black soybeans (*Glycine max* (L.) Merrill) as affected by seed weight

**DOI:** 10.1038/s41598-020-76985-4

**Published:** 2020-11-17

**Authors:** Yu-Mi Choi, Hyemyeong Yoon, Sukyeung Lee, Ho-Cheol Ko, Myoung-Jae Shin, Myung Chul Lee, On Sook Hur, Na Young Ro, Kebede Taye Desta

**Affiliations:** 1 National Agrobiodiversity Center, National Institute of Agricultural Sciences, Rural Development Administration, Jeonju, 54874 Korea; 2 Rural Development Administration, Jeonju, 54875 Korea; 3 Department of Applied Chemistry, Adama Science and Technology University, 1888 Adama, Ethiopia

**Keywords:** Natural variation in plants, Bioanalytical chemistry, Metabolomics, Natural products

## Abstract

Seed weight is regulated by several genes which in turn could affect the metabolite contents, yield, and quality of soybean seeds. Due to these, seed weight is receiving much attention in soybean breeding. In this study, seeds of 24 black soybean varieties and a reference genotype were grown in Korea, and grouped as small (< 13 g), medium (13–24 g), and large (> 24 g) seeds based on their seed weight. The contents of six anthocyanins, twelve isoflavones, and total phenolic, and the antioxidant activities were determined, and the association of each with seed weight was analyzed. The total anthocyanin (TAC) and total isoflavone (TIC) contents were in the ranges of 189.461–2633.454 mg/100 g and 2.110–5.777 mg/g, respectively and were significantly different among the black soybean varieties. By comparison, the average TAC and TIC were the highest in large seeds than in small and medium seeds while the total phenolic content (TPC) was in the order of small seeds > large seeds > medium seeds. Besides, large seeds showed the maximum 1,1-diphenyl-2-picrylhydrazyl radical (DPPH) scavenging activity, whereas small seeds showed the maximum ferric reducing antioxidant power (FRAP) and 2,2′-azino-bis(3-ethylbenzothiazoline-6-sulfonic acid) radical (ABTS) scavenging activities. FRAP activity was positively associated with TIC and TAC, the former association being significant. On the other hand, ABTS and DPPH activities were positively correlated to TPC, the later association being significant. Overall, our findings demonstrated the influence of seed weight on anthocyanin, isoflavone, and phenolic contents and antioxidant activities in black soybeans. Besides, the dominant anthocyanins and isoflavones were the principal contributors to the variations observed in the black soybean varieties, and hence, these components could be selectively targeted to discriminate a large population of black soybean genetic resources.

## Introduction

Soybeans (*Glycine max* (L.) Merr.) are one of the top five largely produced crops in the world, and their demand in food, pharmaceutical, cosmetic, and biofuel industries is increasing^1,2^. Soybeans are generally differentiated based on the color of their seed coats. Physiological and genetic studies indicated that the color variation is due to the difference in the accumulation of pigment-stimulating metabolites^3^. Black soybeans are characterized by their black seed coat color, and they have been widely consumed in Asian countries including Korea, Japan, and China owing to their health benefits^4,5^. Several studies verified that the health benefits of soybeans, in general, are primarily associated with polyphenols mainly of anthocyanins, isoflavones, and phenolic acids. These compounds are proven to have distinct as well as synergistic roles in preventing many ailments such as cancer, diabetes, inflammation, and cardiovascular diseases^6,7^. Moreover, they are considered as the most effective natural antioxidants and manage the over-production of reactive oxygen species (ROS) and reactive nitrogen species (RNS) in the human body via radical-scavenging mechanism^8^.

Many metabolomics studies revealed that anthocyanins are highly concentrated in black soybeans than in other colored soybeans^5,9,10^. These studies also indicated that the glucoside derivatives of cyanidin, delphinidin, and petunidin are the major anthocyanins while other aglycones including pelargonidin, malvidin, and peonidin along with their glucoside and galactoside derivatives are taken as minor, but important components. It is thought that more than 99% of the total anthocyanin in black soybeans is concentrated in the epidermal palisade layer of their seed coat, and contributes to the black pigmentation^11^. Notably, the high level of anthocyanin on top of phenolic content contributes to the greater pharmacological capacities of black soybeans than yellow, green, brown, and other colored soybeans^4,12,13^.. Similar investigations revealed the presence of 12 common isoflavones in soybeans, in general, and they are classified as aglycones (daidzein, glycitein, and genistein), *β*-glycosides (daidzin, glycitin, and genistin), 6″-*O*-malonylglycosides (malonyldaidzin, malonylglycitin, and malonylgenistin), and 6″-*O*-acetylglycosides (acetyldaidzin, acetylglycitin, and acetylgenistin) based on their structures^14–16^. Unlike phenolic compounds and anthocyanins, however, some studies claim that not all of these isoflavones are equally concentrated in black soybeans as they are in other colored soybeans^9,17–19^.

The compositions and contents of polyphenols in plants, in general, are affected by both environmental and genetic factors. Many researchers studied the effect of these factors on the contents of anthocyanins, isoflavones, and phenolic compounds in different soybean varieties including black soybeans^20–24^. Different studies have been targeting different components with respect to both number and identity, and hence, the reported total contents of these polyphenols are wide-ranging. Seed weight is becoming an important factor in these regards and received researchers’ attention. Recent Quantitative Trait Locus (QTL) investigations identified various molecular and physiological mechanisms underlying seed weight differences in soybeans^25–27^. Furthermore, these studies verified that several genes regulate seed weight during development, the variations of which in turn could affect the metabolite compositions, yield, and quality of soybeans. Due to these, studies that assess the correlation between seed-related agronomical characters, polyphenol contents, and their pharmacological activities are becoming of great importance to identify black soybean varieties with high health benefits^11,28^. Moreover, they are applied in the development of improved soybean cultivars, and therefore, are continually desired^29,30^.

In Korea, black soybeans are classified as small, medium, and large seeds based on their 100-seed weights. The choice of black soybeans for consumption by local people also varies based on their seed size. Small seeds are favored for soybean sprouts, while medium and large seeds are preferred to augment vegetables, cooked rice, and home-made fermented pastes^31,32^. Numerous studies have been conducted on the quantitation of anthocyanin, isoflavone, and total phenolic contents in Korean black soybeans. Similarly, many pharmacological studies have been reported^9,10,33–35^. However, most of these studies focused on separate investigations or analyzed only a few numbers of individual polyphenols. Besides, studies that encompass the associations of isoflavone, anthocyanin, and phenolic contents, and biological activities with seed-related characters mainly of seed weight are still limited. The aim of this study was to comprehensively determine the contents of six anthocyanins (cyanidin-3-*O*-glucoside (C-3-*O*-G), delphinidin-3-*O*-glucoside (D-3-*O*-G), petunidin-3-*O*-glucoside (Pt-3-*O*-G), cyanidin-3-*O*-galactoside (C-3-*O*-Ga), peonidin-3-*O*-glucoside (P-3-*O*-G), and malvidin-3-*O*-glucoside (M-3-*O*-G)), 12 isoflavones (daidzein, genistein, glycitein, daidzin, glycitin, genistin, malonylglycitin, malonyldaidzin, malonylgenistin, acetyldaidzin, acetylglycitin, and acetylgenistin), and total phenolic, and the antioxidant activities (ferric reducing antioxidant power (FRAP), 1,1-diphenyl-2-picrylhydrazyl (DPPH) radical scavenging activity, and 2,2′-azino-bis(3-ethylbenzothiazoline-6-sulfonic acid) (ABTS) radical scavenging activity) of black soybean varieties recently grown in Korea, and examine their associations with seed weight.

## Results and discussion

### Plant characteristics

**Figure 1:**
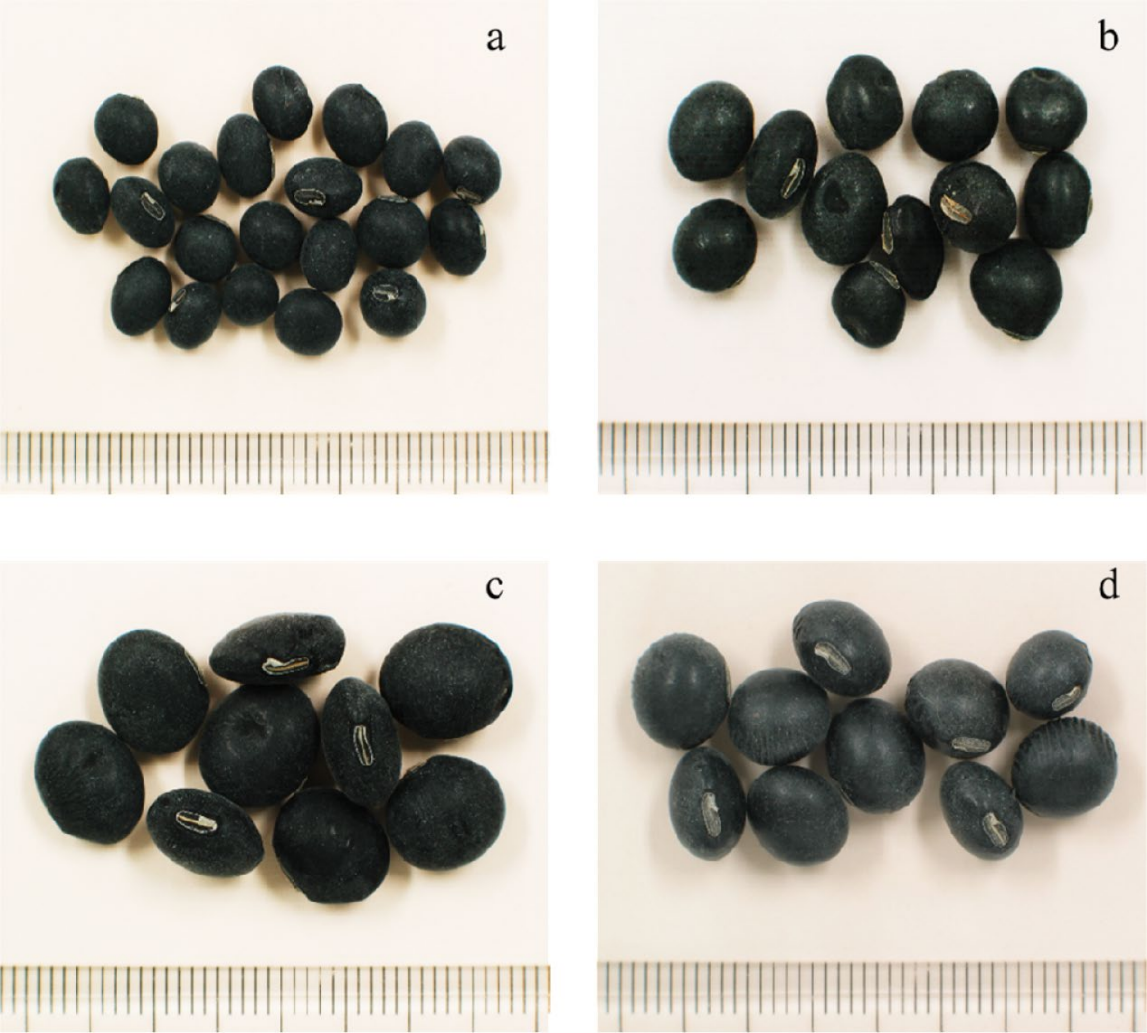
Representative seed samples of the black soybean varieties. Small seeds (**a**), medium seeds (**b**), large seeds (**c**), and Cheongja 2 (**d**).

Variations in agronomical traits are anticipated in soybeans, in general, during their growth period mainly due to differences in variety and genotype^19,30^. The recorded qualitative and quantitative agronomical data for the studied black soybean varieties are summarized in Supplementary Table S1. Cheongja 2, a widely cultivated black soybean variety in Korea owing to its early maturity, yield, and rich metabolite contents, was used as a control^9^. In their growth habit, 50% of the soybean varieties were compact like the control variety while the remaining were semi-spread. Furthermore, all had light purple flowers except BS2 and BS15 that showed white flowers, and BS1, BS4, and BS6 that showed dark purple flowers. Of the entire soybeans, 20 varieties showed brown pod colors, the control variety and BS2 showed light brown pod colors, and three other varieties including BS13, BS14, and BS23 showed mixed pod colors. Each of the black soybean varieties possessed a black hilum color and a brown pubescence pod color. Variety BS2 was the earliest to flower and took 38 days to flowering while BS6 was the latest taking 70 days to flowering. Only BS2 and BS24 flowered earlier than the control variety (48 days) while BS11 and BS4 took equal days. The days to maturity ranged from 108 days for BS2 to 157 days for BS19. Once again, only two varieties, BS2 and BS11, matured earlier than the control variety (125 days). On the other hand, the size of matured seeds was determined in terms of one-hundred seeds weight (HSW) as described before^31^, and the soybeans were classified as small (< 13 g), medium (13–24 g), and large (> 24 g) seeds (Fig. 1). Overall, the majority of the observed characters were shared by other black soybean varieties previously grown in Korea^20,21^.

### Variations in anthocyanin and isoflavone distributions

The distributions of the six anthocyanins and the twelve isoflavones in the black soybean varieties were determined by comparing the retention times of the corresponding standards in the HPLC–DAD chromatograms (Supplementary Figs. S1, S2). The results showed that the target components were not equally distributed among the black soybean varieties (Supplementary Table S2, S3). Among the entire group of soybean varieties, the control variety (Cheongja 2) and seven others including BS5, BS13, BS14, BS17, BS18, BS23, and BS20 contained all the 6 anthocyanins (D-3-*O*-G, C-3-*O*-Ga, C-3-*O*-G, Pt-3-*O*-G, P-3-*O*-G, and M-3-*O*-G). BS2 was atypically different as it presented C-3-*O*-G as the only anthocyanin. Furthermore, nine varieties contained five anthocyanins, six varieties contained four anthocyanins, and one variety contained three anthocyanins. Among the six anthocyanins, C-3-*O*-G, D-3-*O*-G, and Pt-3-*O*-G were the major components and detected in all varieties except in BS2 and BS15. Besides, M-3-*O*-G was the least distributed anthocyanin and detected only in eight varieties. C-3-*O*-Ga and P-3-*O*-G were detected in 19 and 21 varieties, respectively. Overall, the finding was consistent with previous studies where C-3-*O*-G, D-3-*O*-G, and Pt-3-*O*-G were present as the major anthocyanins in seed coats of black soybeans^9,10,36^.

The distribution of target isoflavones among the black soybean varieties was similarly determined using the corresponding external standards (Supplementary Table S3). All the 12 isoflavones (daidzein, genistein, glycitein, daidzin, glycitin, genistin, malonylglycitin, malonyldaidzin, malonylgenistin, acetyldaidzin, acetylglycitin, and acetylgenistin) were present in the studied soybeans although their distribution varied among different varieties. Genistein, glycitin, genistin, daidzin, malonyldaidzin, and malonylgenistin were the most distributed isoflavones and detected in every variety. BS17 was the only exception in this regard because glycitin was not detected in it. Acetylglycitin was the least distributed isoflavone as it was detected only in BS21. Glycitein was also detected only in two varieties including BS21, and BS22, and acetyldaidzin in three varieties including BS1, BS11, and BS21. BS21 was the richest variety in terms of isoflavone diversity and contained all 12 isoflavones except malonylglycitin. Moreover, eight varieties presented nine isoflavones, four varieties including the control variety contained eight isoflavones, and seven varieties presented seven isoflavones. Meanwhile, five varieties including BS3, BS4, BS5, BS13, and BS18 contained only six isoflavones. Many studies also noted differences in isoflavone distributions in black and other colored soybeans including yellow, green, and brown soybeans. For instance, acetylglycitin and acetyldaidzin were not contained in green, brown, and black soybeans grown in Serbia and Korea^9,17^. In addition, Akitha-Devi et al.^37^ did not detect glycitein in yellow soybean cultivars from India. Other than environmental factors, earlier studies directed that structural difference among the isoflavones, and the abundance of specific enzymes could affect the bioavailability and stability of individual isoflavones in soybeans^30,38,39^.

### Variation in anthocyanin content

**Figure 2:**
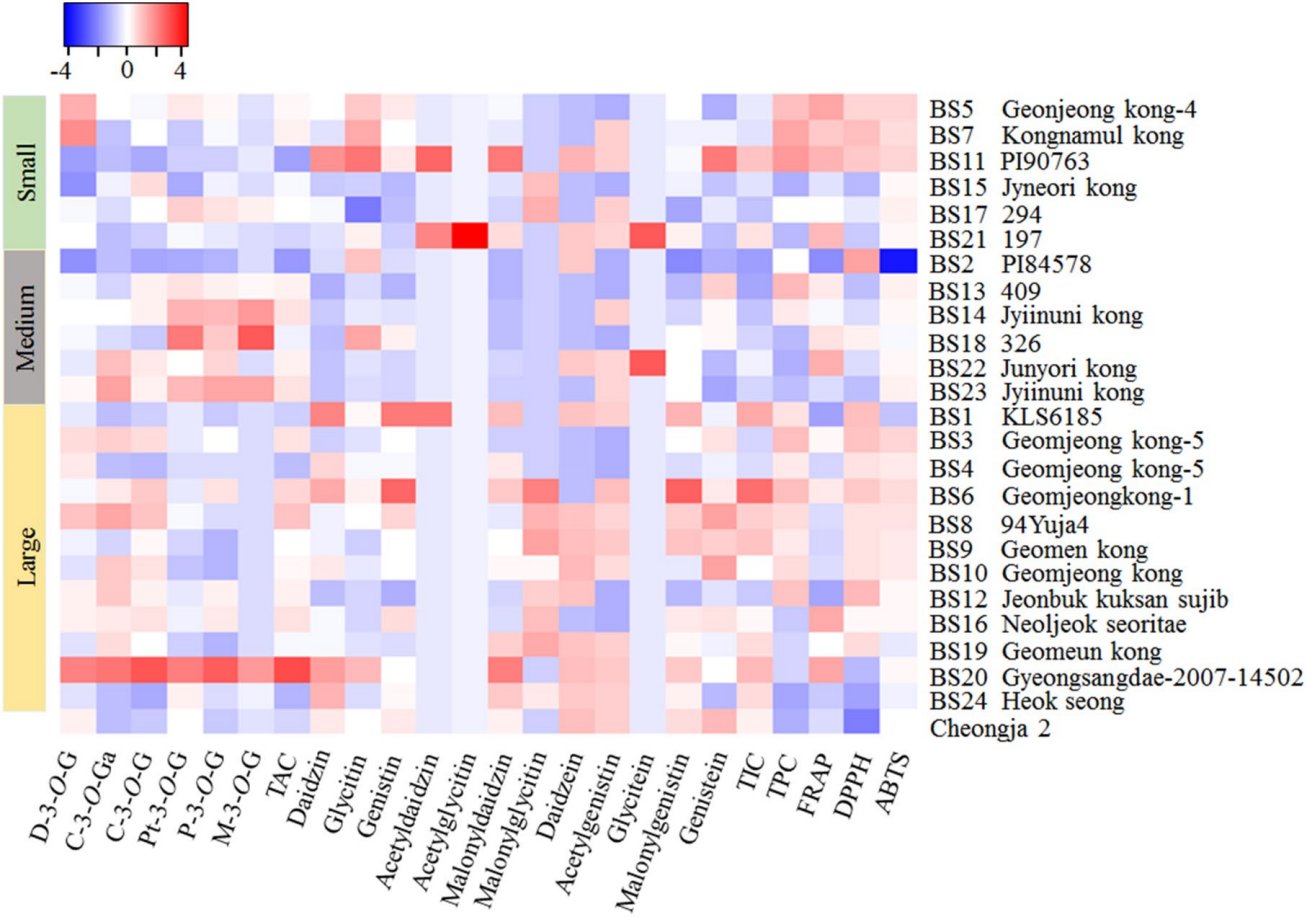
Heatmap showing the relative abundance of six anthocyanins, twelve isoflavones, and total phenolic contents, and antioxidant activities of the studied black soybean varieties. Means are represented by colors ranging from blue for minimum values to red for maximum values. *ABTS* ABTS-radical scavenging activity; *C-3-O-G* cyanidin-3-*O*-glucoside; *C-3-O-Ga* cyanidin-3-*O*-galactoside; *DPPH* DPPH-radical scavenging activity; *D-3-O-G* delphinidin-3-*O*-glucoside; *FRAP* ferric reducing antioxidant power; *M-3-O-G* malvidin-3-*O*-glucoside; *P-3-O-G* peonidin-3-*O*-glucoside; *Pt-3-O-G* petunidin-3-*O*-glucoside; *TAC* total anthocyanin content; *TIC* total isoflavone content; *TPC* total phenolic content.

The contents of individual anthocyanins were determined using peak-area responses of the corresponding external standards in the HPLC–DAD chromatograms. The relative abundances of the six anthocyanins in the studied black soybean varieties are displayed in the heat map in Fig. 2 where means are represented by colors ranging from blue for minimum values to red for maximum values. The corresponding numerical values are obtainable in Supplementary Table S2. The coefficient of variation (CV) ranged from 49.46% in D-3-*O*-G to 197.82% in M-3-*O*-G indicating significant content variations among the black soybeans. With a mean of 990.126 mg/100 g, the total anthocyanin content (TAC) ranged from 189.461 in BS2 to 2633.454 mg/100 g in BS20. The TAC in BS20 was approximately 14 times higher than the TAC in BS2 (p < 0.05). Only six among the entire varieties had lower TAC than the TAC of the control variety (Cheongja 2, 702.00 mg/100 g).

The contents of individual anthocyanins were also significantly different among the soybean varieties. The average D-3-*O*-G, C-3-*O*-Ga, C-3-*O*-G, Pt-3-*O*-G, P-3-*O*-G, and M-3-*O*-G in the entire population were 164.528, 5.682, 725.858, 73.659, 13.774, and 6.850 mg/100 g, respectively. Variety BS20 was typically rich in every anthocyanin except for M-3-*O*-G and hence, could be a potential commercial cultivar in a future agricultural system. The content of C-3-*O*-G was the highest in every variety followed by D-3-*O*-G, and Pt-3-*O*-G although the content of each varied among different varieties. Previous studies reported wide-ranging TAC in black soybeans due to differences in sample type, and the number and identity of targeted anthocyanins^9,10,36^. The results of the current study were comparable to the finding by Zhang et al.^11^ who similarly analyzed these six anthocyanins in seed coats of 60 black soybeans grown in China, and found a TAC between 98.80 and 2132.50 mg/100 g.

The influence of seed weight on the contents of anthocyanins was also assessed and variations were observed among small (< 13 g HSW), medium (13 − 24 g HSW), and large (> 24 g HSW) seeds (Table 1).

**Table 1:** Comparison of individual and total anthocyanin contents in black soybeans of different seed weight.

Seed size	Values (mg/100 g)	D-3-*O*-G	C-3-*O*-Ga	C-3-*O*-G	Pt-3-*O*-G	P-3-*O*-G	M-3-*O*-G	TAC
Small (N = 6)	Max	336.287	5.845	924.025	111.644	19.334	10.680	1085.895
	Min	0.000	0.000	214.281	0.000	4.635	0.000	280.160
	Mean	158.414	2.158	622.907	56.429	12.201	2.404	854.504
Medium (N = 6)	Max	177.706	14.447	856.845	206.707	31.171	53.127	1191.028
	Min	0.000	0.000	189.461	0.0000	16.993	0.000	189.461
	Mean	131.088	5.892	651.324	107.150	20.036	20.209	935.699
Large (N = 12)	Max	359.101	19.824	1968.537	202.127	52.635	31.231	2633.454
	Min	128.936	0.000	229.153	27.471	0.000	0.000	457.508
	Mean	182.388	7.999	838.648	65.647	11.61	2.901	1109.162
Cheongja 2		187.542	0.090	437.358	72.254	3.984	0.772	702.000
CV (%)		50.00	99.49	49.46	69.25	84.81	197.82	45.47
LSD_(0.05)_		50.41	3.33	159.49	17.01	5.00	5.51	219.44

*C-3-O-G* cyanidin-3-*O*-glucoside; *C-3-O-Ga* cyanidin-3-*O*-galactoside; *D-3-O*-G delphinidin-3-*O*-glucoside; *M-3-O-G* malvidin-3-*O*-glucoside; *P-3-O-G* peonidin-3-*O*-glucoside; *Pt-3-O-G* petunidin-3-*O*-glucoside; *TAC* total anthocyanin content.

The TAC was in the ranges of 280.160–1085.895 mg/100 g in small seeds, 189.461–1191.028 mg/100 g in medium seeds, and 457.508–2633.454 mg/100 g in large seeds. In contrast, the average D-3-*O*-G and C-3-*O*-G contents were the highest in large seeds than in small and medium seeds. On the other hand, the average Pt-3-*O*-G, P-3-*O*-G, and M-3-*O*-G contents were the highest in the medium-sized seeds. Small seeds displayed the lowest average content of all anthocyanins except for D-3-*O*-G and P-3-*O*-G contents. In general, the average TAC value was the highest in large seeds (1109.162 mg/100 g) than in medium seeds (935.699 mg/100 g), and small seeds (854.504 mg/100 g). Being the most abundant anthocyanin, C-3-*O*-G accounted for 72.90% of the TAC in small seeds, 69.61% in medium seeds, and 75.61% in large seeds. Kim et al.^31^ also reported a 76% contribution of C-3-*O*-G to TAC in a black soybean variety. On the other hand, Zhang et al.^11^ found as high as 94.1%, and as low as 48.8% contribution of C-3-*O*-G to TAC. Such variations of individual anthocyanin contents in black soybeans could arise due to differences in cultivars, growing conditions, and season^40^. To the best of our knowledge, there is no other study that analyzed the variation of anthocyanins with respect to seed size in black soybeans. Our results suggest that seed weight could be an important agronomical parameter during genotype identification of black soybeans in terms of anthocyanin contents.

### Variation in isoflavone content

The contents of the 12 target isoflavones were determined using peak-area responses of the corresponding external standards, and the results are presented in Fig. 2 and Supplementary Table S3. The black soybean varieties showed significant variation (p < 0.05) with regard to isoflavone contents. With a mean of 2.711 mg/g, the total isoflavone content (TIC) ranged from 2.110 to 5.777 mg/g. The highest TIC was found in BS6 followed by BS1 (4.618 mg/g) and BS20 (4.340 mg/g). BS6 was the variety that had the third-largest TAC (1264.466 mg/100 g) while BS20 was the variety that had the highest TAC (2633.454 mg/100 g). The lowest TIC was found in BS2, which also displayed the lowest TAC (189.461 mg/100 g). Eight varieties including BS1, BS6, BS8, BS9, BS11, BS19, BS20, and BS24 contained higher TIC than the control variety (Cheongja 2, 3.701 mg/g). Overall, some varieties such as BS6, BS20, and BS21 were found to offer a much better isoflavone and anthocyanin contents and compositions than the control variety, and hence, could be developed as commercial cultivars.

The TIC in the present study was comparable with many previous reports although the observed ranges were inconsistent. For instance, Wu et al.^24^ investigated sixteen black soybeans grown in China and found a TIC that ranged from 2.276 to 7.258 mg/g with an average of 4.182 mg/g. Furthermore, Bursać et al.^17^ studied 20 soybean varieties of different seed coat colors grown in Serbia and found an average TIC of 2.760 mg/g in black soybeans. In another study, a TIC as high as 3.580 mg/g was reported in black soybeans grown in Serbia although the isoflavones were quantified in terms of the aglycone concentrations^41^. In a comparable study, Lee et al.^19^ found a much lower average TIC (~ 0.704 mg/g) in black soybeans grown in Korea. These observations signify that the isoflavone contents in black soybeans are wide-ranging. Yet again, differences in cultivars, environment, and analysis protocols could cause such variations^42^. With respect to individual isoflavones, the content of malonylgenistin was the highest in all the studied varieties followed by the content of malonyldaidzin. Previously, Ha et al.^43^, Kim et al.^31^, Lee et al.^19^, and Xu and Chang^36^ also observed high content of malonylgenistin. In other studies, the high content of malonyldaidzin was reported^17,24,31^. These studies disclosed the dominance of malonylgenistin and malonyldaidzin in raw soybean seeds. In contrast, glycitin, acetyldaidzin, and acetylglycitin were the least present isoflavones. This finding was consistent with previous investigations even though the contents were wide-ranging^17,44^. Generally, the total malonylglycoside content was higher in all the black soybeans and contributed between 68.50 and 86.65% of the TIC while the total acetylglycoside content was the lowest and contributed between 1.88 and 8.36% of the TIC as noted also in previous literature^19,24,36^.

The influence of seed weight on the contents of isoflavones was similarly assessed and variations were observed among the three classes (Table 2). The TIC in small, medium and large seeds was in the ranges of 2.734–4.203, 2.110–3.292, and 2.825–5.777 mg/g, respectively.

**Table 2:** Variation of individual and total isoflavone contents in black soybeans of different seed weight.

Isoflavone class	Individual isoflavone	Values (mg/g)										CV (%)	LSD_(0.05)_
		Small seeds (N = 6)			Medium seeds (N = 6)			Large seeds (N = 12)			Cheongja 2		
		Max	Min	Mean	Max	Min	Mean	Max	Min	Mean			
Aglycones	Daidzein	0.138	0.000	0.128	0.117	0.000	0.116	0.131	0.000	0.121	0.128	5.93	0.49
	Glycitein	0.156^a^	0.000	0.156	0.154^a^	0.000	0.154	nd	nd	nd	nd	86.61	1.06
	Genistein	0.136	0.123	0.127	0.130	0.123	0.126	0.133	0.124	0.128	0.131	2.59	0.29
	Total	0.397	0.123	0.195	0.393	0.123	0.190	0.264	0.127	0.209	0.259	41.48	0.57
*β*-glycosides	Daidzin	0.323	0.068	0.143	0.088	0.022	0.054	0.353	0.043	0.179	0.170	65.81	1.34
	Glycitin	0.195	0.000	0.129	0.151	0.075	0.100	0.136	0.068	0.089	0.097	32.32	0.56
	Genistin	0.289	0.160	0.224	0.275	0.152	0.209	0.522	0.143	0.293	0.285	34.74	1.41
	Total	0.808	0.291	0.475	0.472	0.248	0.364	0.941	0.26	0.561	0.552	37.18	3.02
Acetylglycosides	Acetyldaidzin	0.150	0.000	0.133	nd	nd	nd	0.128^a^	0.000	0.128	nd	68.14	1.43
	Acetylglycitin	0.113^a^	0.000	0.113	nd	nd	nd	nd	nd	nd	nd	–	–
	Acetylgenistin	0.096	0.000	0.094	0.096	0.000	0.093	0.108	0.000	0.096	0.096	5.07	0.38
	Total	0.321	0.000	0.189	0.096	0.000	0.093	0.222	0.000	0.112	0.096	55.19	0.49
Malonylglycosides	Malonyldaidzin	1.031	0.534	0.675	0.534	0.45	0.487	1.021	0.525	0.689	0.667	24.30	7.12
	Malonylglycitin	0.418	0.000	0.391	nd	nd	nd	0.639	0.000	0.385	nd	48.30	5.23
	Malonylgenistin	2.009	1.269	1.782	1.937	0.993	1.638	3.245	1.468	2.113	2.126	22.13	23.72
	Total	2.877	2.221	2.588	2.471	1.443	2.125	4.649	2.319	3.06	2.793	22.69	30.85
Total isoflavone content (TIC)		4.203	2.734	3.384	3.292	2.11	2.725	5.777	2.825	3.905	3.701	23.06	32.51

^a^Detected only in one variety; *nd* not detected.

By comparison, the average TIC in large seeds (3.905 mg/g) was the highest followed by the average TIC in small (3.384 mg/g), and medium (2.725 mg/g) seeds. The average total aglycone, total *β*-glycosides, total acetylglycoside, and total malonylglycoside contents were 0.195, 0.475, 0.189, and 2.588 mg/g in small seeds, 0.190, 0.364, 0.093, and 2.125 mg/g in medium seeds, and 0.209, 0.561, 0.112, and 3.060 mg/g in large seeds, respectively each except the average total acetylglycoside content, being the highest in large seeds. As anticipated, the average total malonylglycoside content was dominant while the total acetylglycoside content was the lowest in all the three groups. Among the individual isoflavones, the contents of malonylgenistin and malonyldaidzin were significantly high in large seeds than in small and medium seeds along with their respective *β*-glycosides derivatives (Table 2). Generally, the average TIC among the three black soybean groups was in the order of large seeds > small seeds > medium seeds. In a previous study, Kim et al.^31^ also conducted a similar investigation and found a higher average TIC in small soybean seeds. In another study, high TIC was reported in large as well as in small soybean seeds grown at different sites in Korea^32^. However, neither of these studies clearly outlined the color of the analyzed soybeans. In general, significant variations were observed in isoflavone contents among the studied black soybeans varieties, and our findings further denote the importance of seed weight to distinguish black soybeans with respect to isoflavone contents. Furthermore, the TAC in small, medium and large seeds was approximately 2.53, 3.43, and 2.76 fold higher than the TIC, respectively evidencing the dominance of anthocyanin in the seed coats of black soybeans.

### Variations in total phenolic content and antioxidant activities

**Figure 3:**
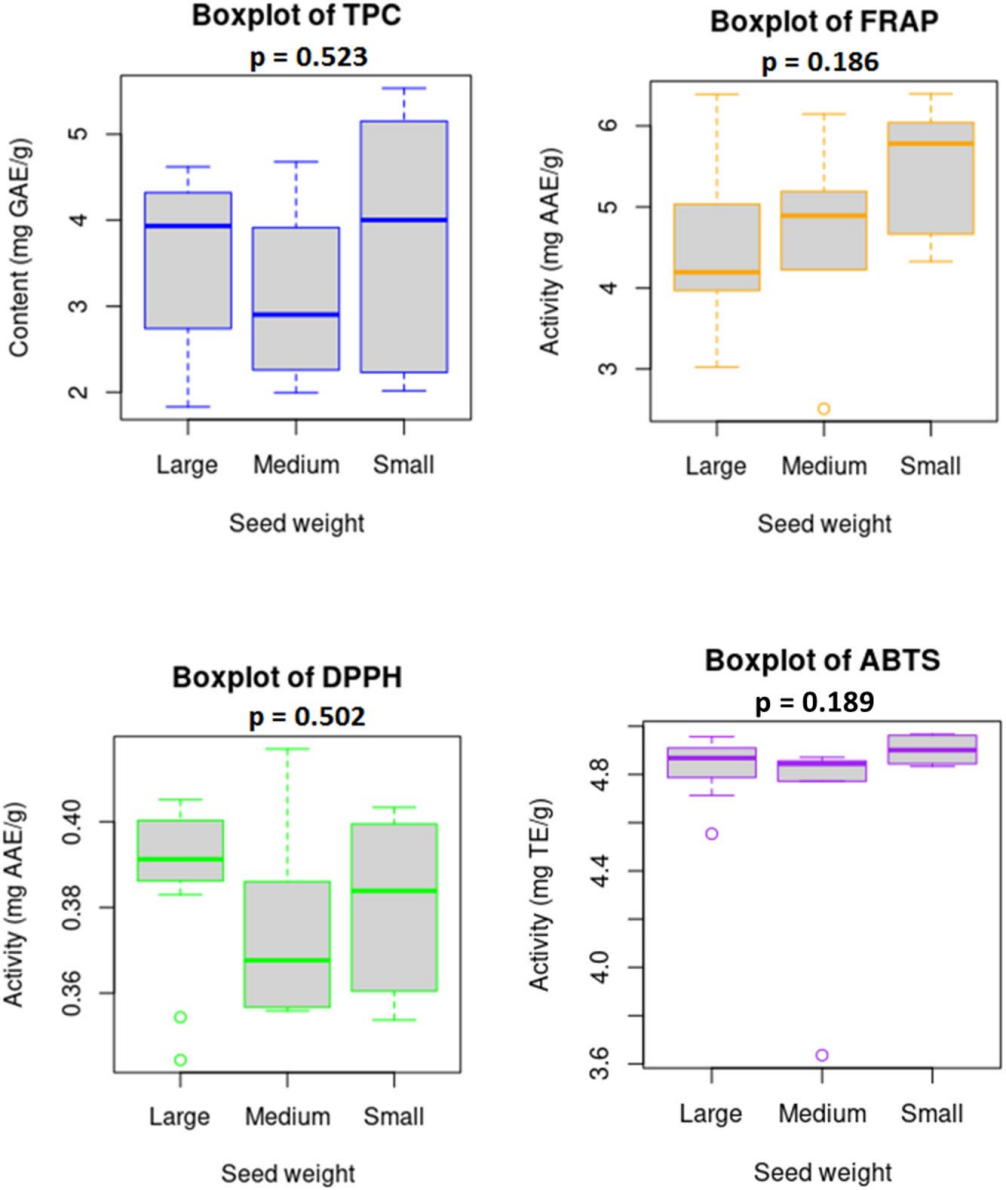
Variability of total phenolic content and antioxidant activities in small, medium, and large seeds of the black soybean varieties. Horizontal lines within boxes represent medians. Bars indicate the maximum and minimum values.

The total phenolic content (TPC) and antioxidant activity results for each of the studied black soybean varieties are presented in Table 3, and their variations with respect to seed weight are displayed by box plots in Fig. 3. With an average of 3.483 mg GAE/g, the TPC ranged from 1.829 mg GAE/g in BS24 to 5.535 mg GAE/g in BS11. The TPC in BS11 was approximately three-fold higher than the TPC in BS24 (*p* < 0.05). Among the studied varieties, 14 soybeans showed higher TPC than the average TPC. Besides, all the black soybean varieties except BS24 showed higher TPC than the control variety (Cheongja 2, 1.987 mg GAE/g). Variety BS20 that had the highest TAC presented a TPC of 2.728 mg GAE/g while variety BS6 that had the highest TIC presented a TPC of 4.621 mg GAE/g. Besides, variety BS2 that contained the lowest TAC and TIC had a TPC of 3.509 mg GAE/g. The TPC was in the ranges of 2.016–5.535, 1.992–4.679, and 1.829–4.621 mg GAE/g in small, medium, and large seeds, respectively (Fig. 2). In general, the average TPC value was in the order of small seeds (3.824 mg GAE/g) > large seeds (3.626 mg GAE/g) > medium seeds (3.108 mg GAE/g), and the result was consistent with previous findings^31^.

**Table 3:** Total phenolic content and antioxidant activities of black soybeans grown in Korea.

Seed size	Variety (sample code)	TPC (mg GAE/g)	Antioxidant activities		
			FRAP (mg AAE/g)	DPPH (mg AAE/g)	ABTS (mg TE/g)
Small (N = 6)	BS5	4.625 ± 0.051^b-d^	6.394 ± 0.115^a^	0.396 ± 0.003^a-d^	4.962 ± 0.007^ab^
	BS15	2.016 ± 0.417^hi^	4.327 ± 0.152^e-g^	0.354 ± 0.019^e-g^	4.844 ± 0.014^d-i^
	BS17	3.382 ± 0.201^e-g^	4.665 ± 0.103^d-f^	0.372 ± 0.020^b-f^	4.853 ± 0.012^c-h^
	BS11	5.535 ± 0.261^a^	6.041 ± 0.263^a-d^	0.399 ± 0.005^a-c^	4.967 ± 0.007^a^
	BS7	5.154 ± 0.185^ab^	5.641 ± 0.330^a-e^	0.403 ± 0.001^ab^	4.950 ± 0.007^a-d^
	BS21	2.230 ± 0.058^hi^	5.920 ± 0.296^a-d^	0.360 ± 0.002^c-g^	4.833 ± 0.004f.^-i^
Medium (N = 6)	BS14	3.916 ± 0.462^c-e^	4.663 ± 0.101^d-f^	0.367 ± 0.012^b-f^	4.837 ± 0.019^e-i^
	BS18	2.258 ± 0.117^hi^	5.191 ± 0.112^a-f^	0.386 ± 0.005 ^a-e^	4.772 ± 0.039^h-j^
	BS13	4.679 ± 0.100^bc^	5.122 ± 0.878^a-f^	0.357 ± 0.001^d-g^	4.856 ± 0.006^b-h^
	BS23	2.294 ± 0.063^hi^	4.224 ± 0.738^fg^	0.356 ± 0.003^d-g^	4.871 ± 0.013^a-h^
	BS22	1.992 ± 0.139^hi^	6.147 ± 0.369^a-c^	0.368 ± 0.003^b-f^	4.852 ± 0.004^c-g^
	BS2	3.509 ± 0.028^ef^	2.510 ± 0.036^h^	0.417 ± 0.007^a^	3.636 ± 0.028^l^
Large (N = 12)	BS20	2.728 ± 0.138^f-h^	6.389 ± 0.115^a^	0.354 ± 0.011^e-g^	4.845 ± 0.005^d-i^
	BS1	3.966 ± 0.077^c-e^	3.024 ± 0.538^gh^	0.403 ± 0.007^ab^	4.554 ± 0.092^k^
	BS6	4.621 ± 0.279^b-d^	5.102 ± 0.280^a-f^	0.399 ± 0.003^a-c^	4.943 ± 0.004^a-e^
	BS4	3.823 ± 0.163^de^	4.111 ± 0.087^fg^	0.389 ± 0.003^a-e^	4.891 ± 0.022^a-g^
	BS24	1.829 ± 0.050^i^	3.842 ± 0.070^f-h^	0.344 ± 0.021^fg^	4.741 ± 0.049^ij^
	BS19	2.753 ± 0.371^f-h^	4.859 ± 0.086^c-f^	0.393 ± 0.008^a-e^	4.714 ± 0.032^j^
	BS12	4.516 ± 0.445^b-d^	3.142 ± 0.101^gh^	0.405 ± 0.006^ab^	4.833 ± 0.022^f-i^
	BS8	4.049 ± 0.173^c-e^	4.187 ± 0.116^fg^	0.391 ± 0.008^a-e^	4.923 ± 0.036^a-f^
	BS16	2.581 ± 0.008^g-i^	6.272 ± 0.156^ab^	0.383 ± 0.011^a-f^	4.846 ± 0.005^d-i^
	BS3	4.613 ± 0.042^b-d^	4.966 ± 0.948^b-f^	0.401 ± 0.009^a-c^	4.956 ± 0.017^a-c^
	BS9	3.902 ± 0.117^c-e^	4.095 ± 0.166^fg^	0.391 ± 0.005^a-e^	4.898 ± 0.018^a-f^
	BS10	4.127 ± 0.259^c-e^	4.201 ± 0.125^fg^	0.391 ± 0.005^a-e^	4.895 ± 0.037^a-g^
Cheongja 2		1.987 ± 0.021^hi^	4.162 ± 0.308^fg^	0.323 ± 0.026^g^	4.787 ± 0.016^g-j^
CV (%)		31.75	22.07	6.10	5.29

Values in the same column marked by different superscript letters are significantly different (p < 0.05).

*TPC* total phenolic content; *FRAP* ferric reducing antioxidant power; *DPPH* DPPH-radical scavenging activity; *ABTS* ABTS-radical scavenging activity.

Previously, many studies examined the TPC in the whole seeds of black soybeans. The TPC value in the present study was much wider than the range reported by Bursać et al.^17^ (2.14–2.39 mg GAE/g) and comparable with the range reported by Kumar et al.^18^ (0.81–5.89 mg GAE/g). Besides, a relatively higher but less wide range was reported by Xu and Chang^40^ (8.75–9.01 mg GAE/g). In addition to the differences in variety and growing conditions, differences in extraction protocols could cause such content variations as noted before.

In vitro antioxidant assays are the method of choice to evaluate the radical scavenging capacities of plant origin phytochemicals. Due to the possible influence of interferences, however, a single assay might not signify the total antioxidant capacity of target phytochemicals. In this study, the antioxidant activities of the black soybeans were estimated in terms of FRAP, DPPH, and ABTS assays (Fig. 3, Table 3). The black soybeans showed a wider variation in their ferric reduction power (CV: 22.07%) than in their radical scavenging capacities. The FRAP activity was in the range of 2.510–6.394 mg AAE/g. Besides, the DPPH and ABTS activities were in the ranges of 0.323–0.417 mg AAE/g and 3.636–4.967 mg TE/g, respectively. Among the studied black soybean varieties, BS5 and BS20 equally displayed the highest FRAP activity while BS11 displayed the highest ABTS activity. BS2, the variety that had the lowest TAC and TIC, displayed the minimum FRAP and ABTS activities. Unlike the FRAP and ABTS activities, BS2 displayed the highest DPPH activity followed by BS12 while the control variety, Cheongja 2, had the lowest DPPH activity. Furthermore, thirteen varieties exhibited a higher DPPH activity than the average DPPH value (0.380 mg AAE/g). These observations suggest the particular contribution of phenolic contents to DPPH-radical scavenging activity. Besides, six varieties including BS2, BS1, BS4, BS24, BS9, and BS12 had a lower FRAP activity than the control variety (4.162 mg AAE/g). Furthermore, five varieties including BS19, BS24, BS1, BS2, and BS18 had lower ABTS value than the control variety (4.787 mg TE g^-1^), and the average value (4.802 mg TE/g). Previously, several studies revealed the antioxidant capacities of black soybeans. However, the difference in assays, sample type, concentration, and result expression made it difficult to compare our findings with those of the previous results^17,40,45^.

With regard to seed weight, the FRAP, DPPH, and ABTS activities were in the ranges of 4.327–6.394 mg AAE/g, 0.354–0.403 mg AAE/g, 4.833–4.967 mg TE/g in small seeds, 2.510–6.147 mg AAE/g, 0.356–0.417 mg AAE/g, 3.636–4.871 mg TE/g in medium seeds, and 3.024–6.389 mg AAE/g, 0.344–0.405 mg AAE/g, and 4.554–4.956 mg TE/g in large seeds, respectively (Fig. 2). Statistical computation did not show significant variation (*p* > 0.05) in any of the antioxidant activities among small, medium, and large seeds (Fig. 3). Nevertheless, small seeds showed a relatively higher average FRAP and ABTS activities than medium and large-sized seeds, whereas large seeds showed higher average DPPH activity.

### Correlation and principal component analysis

**Figure 4:**
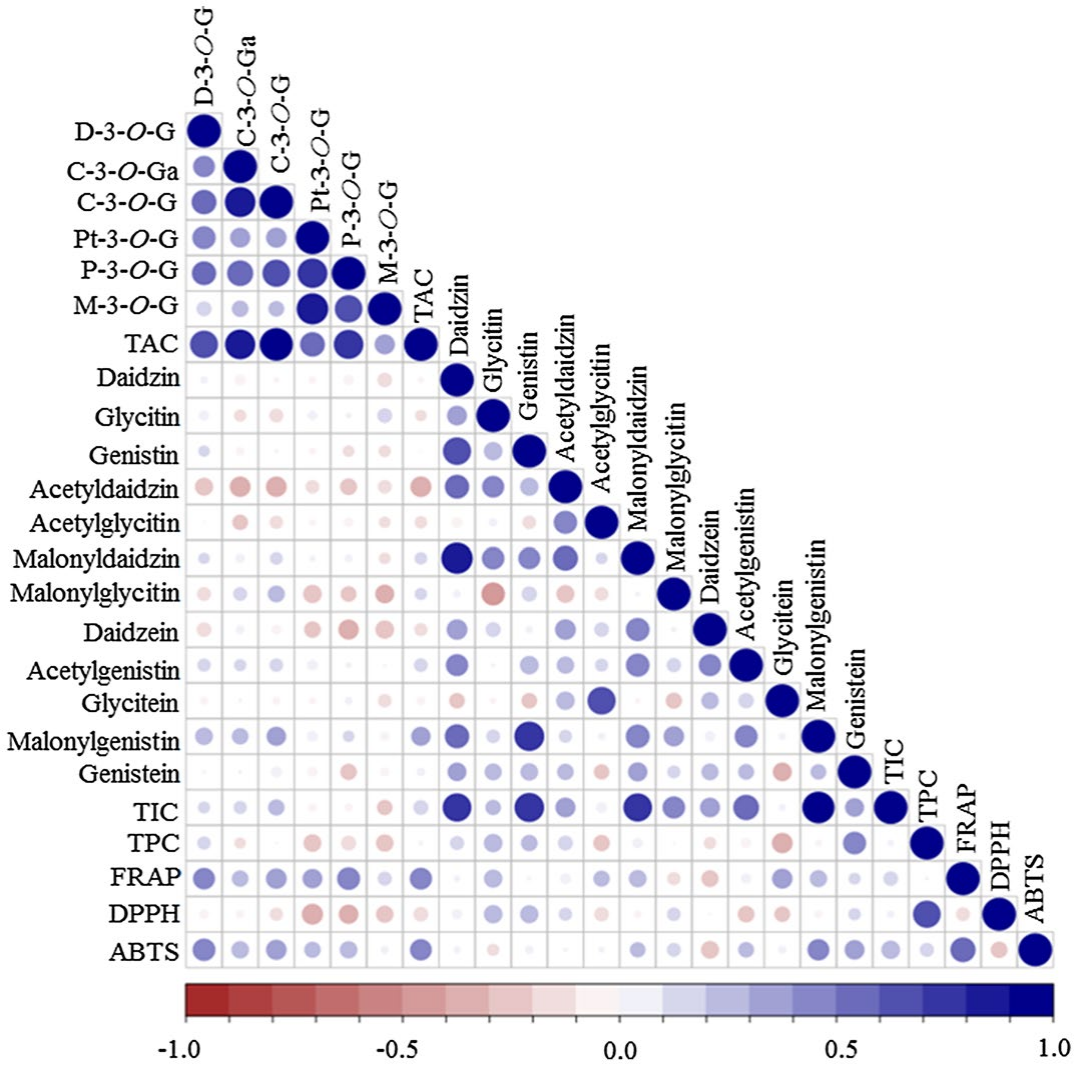
Pearson correlation matrix of the whole data set (isoflavone, anthocyanin, and phenolic contents, and antioxidant activities) obtained from the entire black soybean varieties. *ABTS* ABTS-radical scavenging activity; *C-3-O-G* cyanidin-3-*O*-glucoside; *C-3-O-Ga* cyanidin-3-*O*-galactoside; *DPPH* DPPH-radical scavenging activity; *D-3-O-G* delphinidin-3-*O*-glucoside; *FRAP* ferric reducing antioxidant power; *M-3-O-G* malvidin-3-*O*-glucoside; *P-3-O-G* peonidin-3-*O*-glucoside; *Pt-3-O-G* petunidin-3-*O*-glucoside; *TAC* total anthocyanin content; *TIC* total isoflavone content; *TPC* total phenolic content.

The pair-wise associations between anthocyanins, isoflavones, and total phenolic content, and antioxidant activities were computed using Pearson correlation. The correlation matrix of the entire data set obtained from the whole soybean varieties is displayed in Fig. 4 and the corresponding correlation coefficient (r) values are given in Supplementary Table S4.

All the individual anthocyanins except for M-3-*O*-G, showed positive and significant correlations with TAC (p < 0.05). Likewise, the principal isoflavones including malonyldaidzin, malonylgenistin, and their respective *β*-glycoside derivatives exhibited positive and significant associations with TIC (p < 0.05) while the aglycones and acetylated isoflavones were weakly correlated to TIC. Many of the individual isoflavones showed a negative and/or weak correlations with individual anthocyanins reflecting the trade-off relationship between their biosynthesis pathways^24^. The TAC and TIC were each positively correlated to FRAP and ABTS activities, whereas TPC was significantly correlated with DPPH activity (p < 0.05).

Notably, FRAP and ABTS were strongly correlated with each other (r = 0.551), and each was poorly correlated to DPPH. The observed findings in our study were comparable with many of the previous studies where remarkable associations of anthocyanin, isoflavone, and phenolic extracts with antioxidant activities and metal-reducing capacities were noticed^11,17,28,40^.

**Figure 5:**
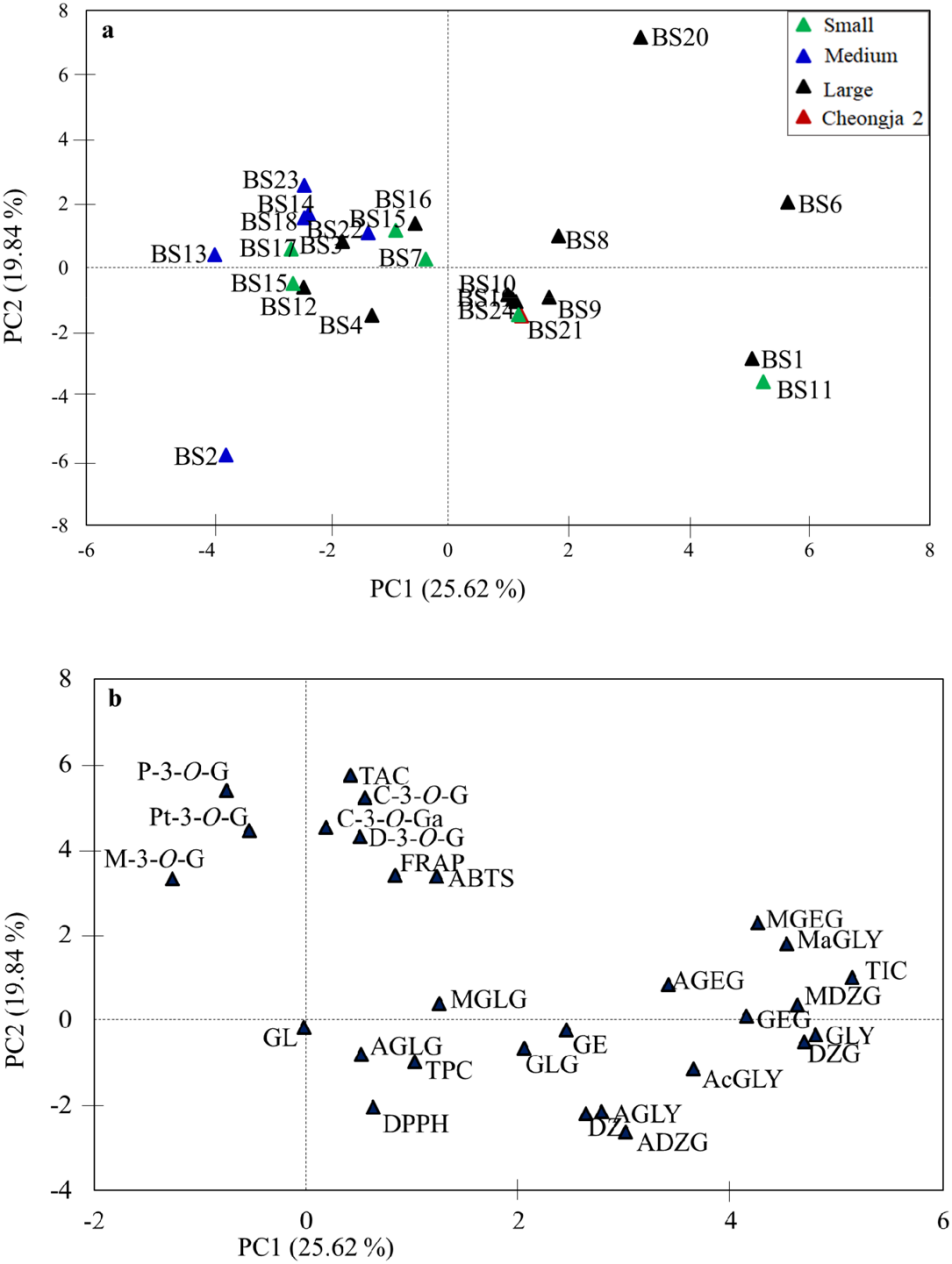
Score plot (**a**) and loading plot (**b**) of the first two principal components obtained from principal component analysis using anthocyanin, isoflavone, and total phenolic contents, and antioxidant activity data of the entire black soybean varieties. *AcGLY* total acetylglycoside content; *AGLY* total aglycone content *ADZG* acetyldaidzin; *AGEG* acetylgenistin; *AGLG* acetylglycitin; *DZ* daidzein; *DZG* daidzin; *GE* genistein; *GEG* genistin; *GL* glycitein; *GLY* total *β*-glycoside content; *GLG* glycitin; *MaGLY* total malonylglycoside content; *MDZG* malonyldaidzin; *MGEG* malonylgenistin; *MGLG* malonylglycitin. Other abbreviations represent similar descriptions as in Fig. 4.

The distribution of the black soybean varieties and their association with isoflavone, anthocyanin, and total phenolic contents, and antioxidant activities were further viewed by principal component analysis (PCA). The PCA yielded seven components with eigenvalues > 1. The first two components (PC1 and PC2) alone explained about 45.46% of the cumulative variance and hence, were considered for analysis (Fig. 5).

The isoflavone contents were the major discriminant factors for the variability observed along PC1 while anthocyanin contents, FRAP, and ABTS were the contributors for the variability observed along PC2. The most abundant isoflavones including malonyldaidzin (9.90%) and malonylgenistin (8.36%) together with daidzin (10.18%) were the major contributors along PC1. Besides, all the anthocyanins except M-3-*O*-G contributed ≥ 8.5% to the variance along PC2 while TPC and DPPH were negatively associated with it. These observations suggest that components that showed strong associations with their respective total contents and contributed more to variations observed along PC1 and PC2 could selectively be targeted when assessing a large population of soybean genetic materials. Overall, the first two principal components were able to discriminate black soybeans with high TAC (BS20, BS8, BS6) and TIC (BS1, BS11, and BS6) at a 45.46% cumulative variance. In addition, the black soybean variety that had the lowest TAC and TIC (BS2) was displayed on the negative sides of both PC1 and PC2 (Fig. 5a). Remarkably, the positive associations between TAC and FRAP as well as TPC and DPPH were also noticeable in the loading plot (Fig. 5b).

## Conclusion

The primary objective of this study was to comprehensively determine the anthocyanin, isoflavone, and total phenolic contents, and antioxidant activities in black soybean varieties and assess the influence of seed weight on each. Statistical analysis showed significant variations among the studied varieties with respect to both contents and antioxidant activities. Variations were also observed in anthocyanin, isoflavone, and phenolic contents and antioxidant activities with respect to seed weight. Three anthocyanins including C-3-*O*-G, Pt-3-*O*-G, and D-3-*O*-G, and two isoflavones including malonylgenistin and malonyldaidzin were found to be the foremost components in each of the studied soybean varieties. The anthocyanin and isoflavone contents were the determinant factors to discriminate small, medium and large seeds. In contrast, large seeds were characterized by high total anthocyanin and total isoflavone contents compared to medium and small-sized seeds, whereas small seeds were characterized by high total phenolic contents. The correlation analysis exhibited a strong association of anthocyanin and isoflavone contents with FRAP, and phenolic contents with DPPH and ABTS-radical scavenging activities. Generally, the present study comprehensively showed the variations of the most common anthocyanins and isoflavones, and total phenolic contents and antioxidant activities in black soybean varieties recently grown in Korea. Furthermore, our findings emphasize that seed weight could be a significant agronomical parameter to discriminate large populations of black soybean genotypes with respect to their polyphenol contents and antioxidant activities.

## Materials and methods

### Chemicals and reagents

All the chemicals and reagents used in the present study were of analytical grade (purity > 98.9%). Anthocyanin reference standards including cyanidin-3-*O*-glucoside, cyanidin-3-*O*-galactoside, peonidin-3-*O*-glucoside, and malvidin-3-*O*-glucoside were purchased from PhytoLab (Bavaria, Germany). Isoflavone standards including malonyldaidzin, malonylgenistin acetylgenistin, acetylglycitin, and acetyldaidzin were purchased from Synthose (Ontario, Canada) while malonylglycitin was purchased from Fujifilm (Osaka, Japan). HPLC-grade solvents including water and methanol were purchased from Thermo Fisher (Swedesboro, NJ, USA). Acetonitrile and acetic acid were purchased from Honeywell (Charlotte, NC, USA) and Merck (Darmstadt, Germany), respectively. The remaining reference standards and reagents including delphinidin-3-*O*-glucoside, petunidin-3-*O*-glucoside, daidzein, daidzin, genistein, genistin, glycitein, glycitin, hydrochloric acid (HCl), acetone, ethanol, formic acid, gallic acid, Folin-Ciocalteu phenol reagent, *L*-ascorbic acid, sodium carbonate, potassium ferricyanide, trichloroacetic acid, ferric chloride, diammonium salt of 2,2′-azino-bis(3-ethylbenzothiazoline-6-sulfonic acid) (ABTS), 1,1-diphenyl-2-picrylhydrazyl radical (DPPH∙), and 6-hydroxy-2,5,7,8-tetramethylchromane-2-carboxylic acid (Trolox) were purchased from Sigma Aldrich (St.Louis, MO, USA).

### Plant materials

Seeds of 24 Korean black soybean varieties and a commonly grown commercial genotype in Korea (Cheongja 2) with a specific introduction (IT) number were provided by the Gene Bank of National Agrobiodiversity Center (Jeonju, Korea). The soybean seeds were cultivated under similar conditions during the country’s cropping season (June–November 2019) in an experimental field located at the center. The change in pod color was used as a maturity index, and seeds were harvested when approximately 95% of the pods reached mature color^46^. The collected whole seed samples were dried in Bionex oven (Vision Scientific, Daejon, Korea) for three days at 50 °C, and used for the analysis of isoflavone and total phenolic contents. Besides, seed coat samples were lyophilized in an LP500 freeze drier (ilShinBioBase, Dongducheon, Korea), and used for anthocyanin analysis. Dried whole seed and seed coat samples were powdered through a 315 mesh sieve and stored at -20 °C until extraction. The Rural Development Administration (RDA, Jeonju, Korea) guideline was used to classify seeds as small (< 13 g), medium (13–24 g), and large (> 24 g) based on their one hundred seeds weight (HSW)^31^. For ease of presentation, the black soybean varieties were coded and numbered based on the order of their IT-number. The agronomical character, variety names, and codes are presented in the Supplementary Table S1.

### Extraction of anthocyanin and isoflavone

The extraction of anthocyanin and isoflavone was conducted according to a recently reported method^24^, and the sample and solvent conditions were optimized in our laboratory. For anthocyanin extraction, 0.1 g of powdered seed coat sample was placed into a 15 mL extraction tube which was enfolded with aluminum foil. Then, 4 mL of pre-chilled 80% methanol containing 1% HCl was added, and the mixture was vortexed for 2 min. The mixture was sonicated in an ice bath at 4 °C for 30 min in the dark followed by centrifugation at 4000 rpm for 15 min. The upper supernatant was retained, and the extraction process was repeated one more time for the residue. During isoflavone extraction, 0.5 g of powdered whole seed sample was added into 15 mL extraction tube. Then, 10 mL of 80% methanol was added, and the mixture was vortexed for 2 min followed by sonication in a water bath at 25 °C for 15 min. Likewise, the mixture was centrifuged at 4000 rpm for 10 min, the upper supernatant was pipetted, and the extraction cycle was repeated one more time for the residue. In each case, about 2 mL of the combined supernatant was taken and filtered through a 0.45 μm PTFE membrane into an injection vial for subsequent analysis. HPLC-analysis was conducted within 48 h after the extraction, and the aliquots were stored at -20 °C when not used.

### RP-HPLC–DAD analysis

Identification and quantification of anthocyanins and isoflavones were conducted using a 1260-Infinity Quaternary HPLC system (Agilent Technologies, Santa Clara, CA, USA) which was coupled to a diode-array-detector (DAD). Individual anthocyanins and isoflavones were identified by comparing the retention times of the corresponding external standards in HPLC–DAD chromatograms. For quantification, calibration curves were plotted from peak area responses of each standard at five concentration levels (500, 250, 125, 75, and 25 mg/L for anthocyanins, and 80, 40, 20, 10, and 5 mg/L for isoflavones, with *r*^2^ > 0.999). The anthocyanins and isoflavones were quantified from peak areas of the acquired chromatograms of each sample. Separation of anthocyanins and isoflavones was achieved using a reverse-phase (RP) Inertsil ODS-3 (250 × 4.6 mm, 5 μm) column (GL Sciences, Tokyo, Japan). During anthocyanin analysis, the column was maintained at 35 °C, and a binary solvent system composed of water containing 5% formic acid (A) and a mixture of methanol and acetonitrile (1:1, *v/v*) containing 5% formic acid (B) was used as mobile phase. The gradient condition started with 5% solvent B followed by an increase to 20% for 20 min, to 25% for 15 min, and then to 30% for 5 min. The final condition was maintained isocratic for one minute. During isoflavone analysis, the column was maintained at 30 °C, and the mobile phase was composed of water containing 0.1% acetic acid (A) and acetonitrile (B). The gradient elusion was optimized to start with 18% solvent B followed by an increase to 20% for 20 min, and to 50% for 39 min. Then, the final condition was maintained isocratic for one minute. In each case, the solvent flow rate was 1 mL/min, and the sample injection volume was 0.5 *μ*L throughout the analysis. Anthocyanins were detected at λ_max_ 525 nm while isoflavones were detected at λ_max_ 254 nm. The acquired chromatograms were read and executed using Agilent ChemStation software (Agilent Technologies, Santa Clara, CA, USA).

### Extraction and determination of total phenolic contents

Phenolic compounds were extracted using a previously reported protocol with some modifications^45^. Specifically, 1.0 g of whole seed powder of each sample (in triplicate) was individually placed in a 45 mL extraction tube, mixed with 15 mL of 70% acetone (aqueous), and sonicated for 25 min at 25 °C in dark. The mixture was then taken off, centrifuged at 4000 rpm for 10 min, and the supernatant was recovered. The extraction cycle was repeated one more time for the residue with 5 mL of the solvent. The total phenolic content (TPC) of the pooled supernatant was determined using the Folin-Ciocalteu method against gallic acid standard (Choi et al. 2020). Briefly, 100 μL of the phenolic extract was mixed with an equal volume of Folin-Ciocalteu reagent. The mixture was allowed to react in the dark for 3 min at 25 °C. Then, 100 μL of sodium carbonate solution (Na_2_CO_3_, 2%) was added, and the mixture was incubated for 30 min more. Finally, the absorbance of the mixture was measured (λ_max_ 750 nm) using an Eon Microplate Spectrophotometer (Bio-Tek, Winooski, VT, USA). For each sample, the result was computed from triplicate measurements and expressed as mg gallic acid equivalents per g of dried seed weight (mg GAE/g).

### Antioxidant activities

The antioxidant activities of each phenolic extract obtained from the whole seed samples were determined using three assays including ferric reducing antioxidant power (FRAP), 1,1-diphenyl-2-picrylhydrazyl radical scavenging activity (DPPH), and 2,2′-azino-bis(3-ethylbenzothiazoline-6-sulfonic acid) radical scavenging activity (ABTS). The FRAP activity was determined according to a previously described method with some modification^47^. Briefly, 60 µL of the phenolic extract of each sample was individually mixed with 150 µL of phosphate buffer (pH: 6.6, 0.2 M) and 150 µL of potassium ferricyanide (K_3_Fe(CN)_6_, 1.0%) in a 1.5 mL tube. Each mixture was incubated for 20 min at 50 °C followed by the addition of 150 µL of trichloroacetic acid (10%). The mixture was then centrifuged at 13,000 rpm for 10 min. The upper supernatant was pipetted, and 100 µL of it was diluted with 100 µL of distilled water and 20 µL of ferric chloride solution (0.1%). After 10 min, the absorbance was recorded (λ_max_: 700 nm) using an Eon Microplate Spectrophotometer (Bio-Tek, Winooski, VT, USA). *L*-ascorbic acid was used as standard, and the FRAP activity of each sample was determined as mg ascorbic acid equivalents per g of dried seed weight (mg AAE/g). The DPPH and ABTS activities were conducted according to our recently reported method^48^. The DPPH activity was expressed as mg AAE/g while the ABTS activity was presented as mg Trolox equivalents per g of dried seed weight (mg TE/g).

### Statistical analysis

Measurements were made in triplicate during every analysis unless specified, and results were expressed as mean ± standard deviation (SD). One-way analysis of variance and the least significant difference at a 0.05 probability level (LSD_(0.05)_) were computed using Xlstat-software (Addinsoft, NY, USA) and Minitab-18 software (Minitab Inc., PA, USA), and used to statistically determine the difference between treatments. Heatmap, box plot, and Pearson correlation matrix were schemed using R-software version 4.0.0 (https://www.r-project.org/).

## Supplementary information

Supplementary Information
