# Supplementary material for: Isoflavones, anthocyanins, phenolic content, and antioxidant activities of black soybeans (Glycine max (L.) Merrill) as affected by seed weight
Source: Sci Rep. 2020 Nov 17;10:19960. doi: 10.1038/s41598-020-76985-4 (PMC7673111; doi:10.1038/s41598-020-76985-4)
Supplement: Supplementary file 1 — Supplementary Information [file 41598_2020_76985_MOESM1_ESM.pdf]

Isoflavones, anthocyanins, phenolic content, and antioxidant activities of black soybeans (*Glycine max* (L.) Merrill) as affected by seed weight

Yu-Mi Choi, Hyemyeong Yoon, Sukyeung Lee, Ho-Cheol Ko, Myoung-Jae Shin, Myung Chul Lee, On Sook Hur, Na Young Ro, Kebede Taye Desta

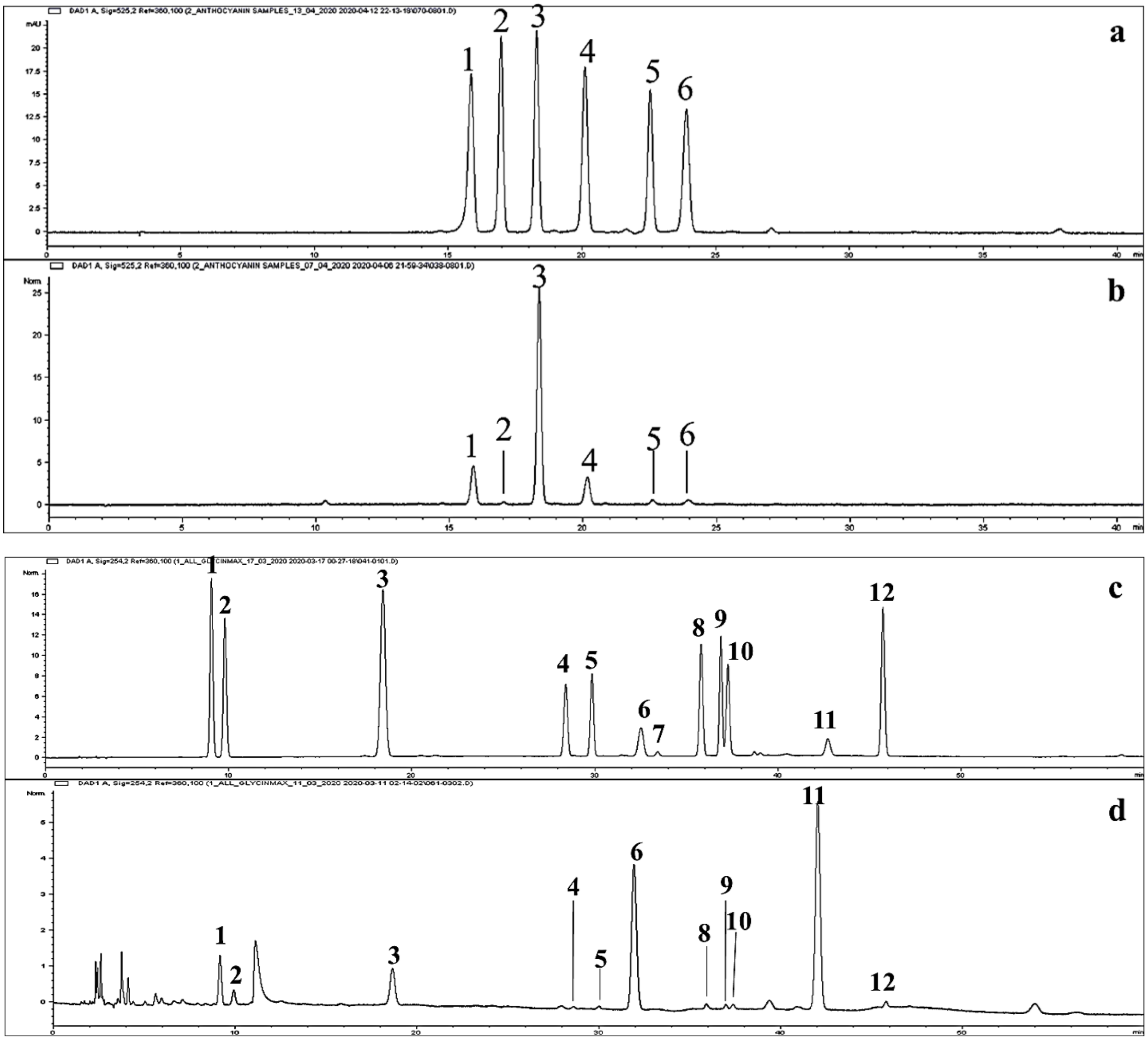

Supplementary Figure S1. HPLC chromatograms of standard anthocyanin mixture (**a**), a representative seed coat sample extract (**b**), standard isoflavone mixture (**c**), and a representative seed sample extract (**d**). Peak assignment (**a** and **b**: **1**. Delphinidin-3-*O*-glucoside, **2**. Cyanidin-3-*O*-galactoside, **3**. Cyanidin-3-*O*-glucoside, **4**. Petunidin-3-*O*-glucoside, **5**. Peonidin-3-*O*-glucoside, **6**. Malvidin-3-*O*-glucoside; **c** and **d**: **1**. Daidzin, **2**. Glycitin, **3**. Genistin, **4**. Acetyldaidzin, **5**. Acetylglycitin, **6**. Malonyldaidzin, **7**. Malonylglycitin, **8**. Daidzein, **9**. Acetylgenistin, **10**. Glycitein, **11**. Malonylgenistin, **12**. Genistein).

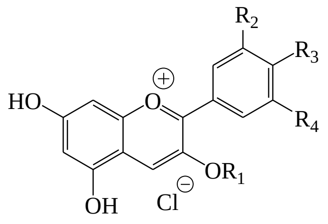

R1	R2	R3	R4	Anthocyanin
Glc	OH	OH	OH	Delphinidin-3-*O*-glucoside
Gal	OH	OH	H	Cyanidin-3-*O*-galactoside
Glc	OH	OH	H	Cyanidin-3-*O*-glucoside
Glc	OCH_3	OH	OH	Petunidin-3-*O*-glucoside
Glc	OCH_3	OH	H	Peonidin-3-*O*-glucoside
Glc	OCH_3	OH	OCH_3	Malvidin-3-*O*-glucoside

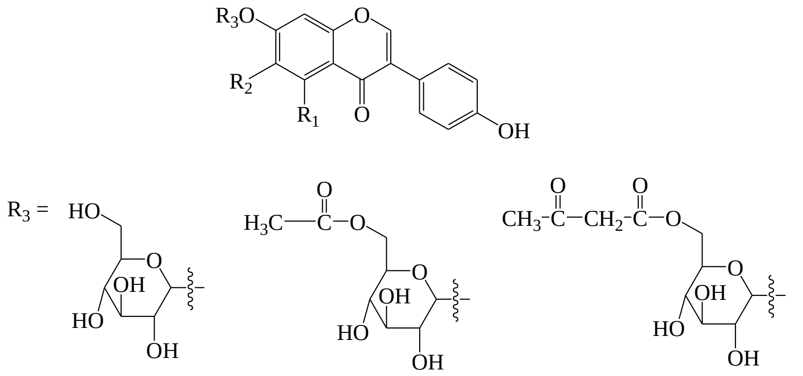

Glucoside (Glc) Acetylglucoside (AcGlc) Malonylglucoside (MaGlc)

R1	R2	R3	Isoflavone
H	H	Glc	Daidzin
H	OCH_3	Glc	Glycitin
OH	H	Glc	Genistin
H	H	AcGlc	Acetyldaidzin
H	OCH_3	AcGlc	Acetylglycitin
H	H	MaGlc	Malonyldaidzin
H	OCH_3	MaGlc	Malonylglycitin
H	H	H	Daidzein
OH	H	AcGlc	Acetylgenistin
H	OCH_3	H	Glycitein
OH	H	MaGlc	Malonylgenistin
OH	H	H	Genistein

Supplementary Figure S2. Chemical structures of individual anthocyanins and isoflavones analyzed in black soybeans.

Supplementary Table S1. Variety names, sample codes and agronomical characteristics of black soybean varieties grown in Korea.

Code	Variety name	IT Number	Hilum color	Flower color	Growth habit	Pod color	Pubescence color	DF (days)	DM (Days)	HSW(g)
BS1	KLS6185	IT143347	Black	Dark purple	Compact	Brown	Brown	51	141	28.1
BS2	PI84578	IT161904	Black	White	Semi-spread	Light brown	Brown	38	108	19.6
BS3	Geomjeong kong-5	IT177271	Black	Light purple	Compact	Brown	Brown	64	143	42.1
BS4	Geomjeong kong-5	IT177573	Black	Dark purple	Compact	Brown	Brown	48	134	34.9
BS5	Geomjeong kong-4	IT177709	Black	Light purple	Compact	Brown	Brown	58	126	9.9
BS6	Geomjeong kong-1	IT178054	Black	Dark purple	Semi-spread	Brown	Brown	70	147	30.0
BS7	Kongnamul kong	IT186183	Black	Light purple	Semi-spread	Brown	Brown	64	141	12.3
BS8	94Yuja4	IT189215	Black	Light purple	Semi-spread	Brown	Brown	64	139	39.6
BS9	Geomen kong	IT194558	Black	Light purple	Semi-spread	Brown	Brown	63	142	43.8
BS10	Geomjeong kong	IT194560	Black	Light purple	Semi-spread	Brown	Brown	58	153	55.0
BS11	PI90763	IT21665	Black	Light purple	Semi-spread	Brown	Brown	48	113	11.8
BS12	Jeonbuk kuksan sujib	IT224192	Black	Light purple	Semi-spread	Brown	Brown	64	148	39.5
BS13	409	IT228822	Black	Light purple	Compact	Mixed	Brown	64	139	14.7
BS14	Jyiinuni kong	IT231544	Black	Light purple	Compact	Mixed	Brown	59	139	13.1
BS15	Jyineori kong	IT239896	Black	White	Semi-spread	Brown	Brown	55	126	11.1
BS16	Neoljeok seoritae	IT252252	Black	Light purple	Compact	Brown	Brown	64	147	39.9
BS17	294	IT252748	Black	Light purple	Compact	Brown	Brown	55	135	11.6
BS18	326	IT252768	Black	Light purple	Semi-spread	Brown	Brown	55	135	13.6
BS19	Geomeun kong	IT263853	Black	Light purple	Semi-spread	Brown	Brown	64	157	38.0
BS20	Gyeongsangdae-2007-14502	IT274515	Black	Light purple	Compact	Brown	Brown	51	132	24.3
BS21	197	IT275005	Black	Light purple	Semi-spread	Brown	Brown	64	126	12.4
BS22	Junyori kong	IT308619	Black	Light purple	Semi-spread	Brown	Brown	64	139	15.1
BS23	Jyiinuni kong	IT311261	Black	Light purple	Compact	Mixed	Brown	64	139	14.7
BS24	Heuk seong	ITK137773	Black	Light purple	Compact	Brown	Brown	43	126	36.0
Cheongja 2			Black	Light purple	Compact	Light brown	Brown	48	125	35.5

DF: Days to flowering; DM: Days to maturity; HSW: One-hundred seeds weight

Supplementary Table S2. Individual and total anthocyanin contents in seed coats of black soybeans grown in Korea.

Seed size	Sample Code	Individual and Total Anthocyanin contents (Mean ± SD, mg/100g)						
		**D-3-*O*-G**	**C-3-*O*-Ga**	**C-3-*O*-G**	**Pt-3-*O*-G**	**P-3-*O*-G**	**M-3-*O*-G**	**TAC**
Small (N = 6)	BS5	274.234 ± 40.827[b]	5.845 ± 0.790[e-g]	686.178 ± 103.350[g]	92.204 ± 13.551[d]	15.692 ± 2.230[f-i]	0.894 ± 0.173[d]	1075.047 ± 37.017[c-g]
	BS15	nd	4.340 ± 0.663[f-h]	924.025 ± 51.211[b-d]	nd	11.269 ± 0.802[i-l]	nd	939.633 ± 23.796[fg]
	BS17	153.980 ± 12.696[e-i]	2.619 ± 1.148[g-i]	735.819 ± 63.656[e-g]	111.644 ± 10.584[c]	19.334 ± 2.891[d-f]	10.680 ± 2.105[c]	1034.077 ± 21.968[d-g]
	BS11	21.965 ± 2.235[j]	nd	214.281 ± 72.436[j]	36.427 ± 7.777[ij]	4.635 ± 0.487[m]	2.851± 0.599[d]	280.160 ± 27.991[lm]
	BS7	336.287 ± 34.220[a]	0.146 ± 0.663[i]	704.332 ± 78.546[fg]	32.560 ± 5.849[ij]	12.570 ± 0.637[h-k]	nd	1085.895 ± 29.999[c-f]
	BS21	164.018 ± 10.732[d-i]	nd	472.743 ± 26.907[h]	65.740 ± 5.236[f]	9.708 ± 2.710[j-l]	nd	712.209 ± 9.413[h-j]
Medium (N = 6)	BS14	162.497 ± 8.777[d-i]	5.200 ± 0.790[e-h]	827.717 ± 50.473[d-g]	136.885 ± 9.072[b]	27.009 ± 6.104[bc]	31.720 ± 1.351[b]	1191.028 ± 17.173[b-d]
	BS18	154.386 ± 8.522[e-i]	2.512 ± 1.714[hi]	424.537 ± 3.780[hi]	206.707 ± 3.913[a]	23.497 ± 3.073[cd]	53.127 ± 2.890[a]	864.766 ± 2.153[g-i]
	BS13	156.008 ± 4.844[e-i]	1.974 ± 0.790[hi]	812.332 ± 15.155[d-g]	96.682 ± 2.076[cd]	16.993 ± 0.487[e-h]	9.212 ± 0.915[c]	1093.201 ± 5.180[c-f]
	BS23	177.706 ± 9.389[d-i]	14.447 ± 2.361[b]	797.050 ± 38.189[d-g]	133.018 ± 3.976[b]	31.171 ± 2.556[b]	27.194 ± 2.249[b]	1180.587 ± 12.941[b-e]
	BS22	135.932 ± 9.892[g-i]	11.222 ± 0.663[bc]	856.845 ± 39.962[d-f]	69.608 ± 3.808[ef]	21.545 ± 1.149[de]	nd	1095.152 ± 14.803[c-f]
	BS2	nd	nd	189.461 ± 15.207[j]	nd	nd	nd	189.461 ± 0.000[m]
Large (N = 12)	BS20	359.101 ± 33.728[a]	19.824 ± 2.503[a]	1968.537 ± 154.059[a]	202.127 ± 16.023[a]	52.635 ± 6.023[a]	31.231 ± 6.769[b]	2633.454 ± 53.566[a]
	BS1	138.771 ± 20.894[f-i]	nd	448.537 ± 60.507[hi]	58.310 ± 7.726[fg]	4.505 ± 0.184[m]	nd	650.124 ± 23.256[i-k]
	BS6	153.473 ± 12.312[e-i]	7.566 ± 1.299[d-f]	1029.153 ± 69.132[bc]	55.461 ± 3.117[f-h]	18.814 ± 2.298[d-g]	nd	1264.466 ± 26.051[bc]
	BS4	190.786 ± 49.764[c-e]	nd	304.332 ± 39.589[ij]	45.791 ± 4.030[g-i]	7.236 ± 0.487[lm]	nd	548.146 ± 21.548[jk]
	BS24	131.471 ± 5.344[hi]	nd	229.153 ± 5.132[j]	85.282 ± 4.347[de]	8.017 ± 0.487[k-m]	3.585 ± 1.668[d]	457.508 ± 1.958[kl]
	BS19	128.936 ± 10.889[i]	8.211 ± 3.053[c-e]	728.640 ± 56.059[e-g]	36.326 ± 3.157[ij]	nd	nd	902.112 ± 22.037[f-h]
	BS12	180.748 ± 5.642[d-h]	9.824 ± 1.097[cd]	817.871 ± 11.216[d-g]	55.257 ± 4.545[f-h]	16.472 ± 0.843[f-h]	nd	1080.172 ± 3.774[c-g]
	BS8	239.253 ± 17.577[bc]	13.695 ± 2.651[b]	1064.845 ± 78.435[b]	64.316 ± 4.693[f]	6.976 ± 0.663[lm]	nd	1389.084 ± 29.416[b]
	BS16	185.514 ± 46.630[d-g]	7.135 ± 1.844[d-f]	899.307 ± 207.135[cd]	59.430 ± 13.760[fg]	18.293 ± 2.238[e-g]	nd	1169.679 ± 78.132[b-e]
	BS3	206.705 ± 28.645[cd]	9.394 ± 2.540[cd]	918.076 ± 118.900[b-d]	57.802 ± 8.272[fg]	14.001 ± 1.288[g-j]	nd	1205.977 ± 44.580[b-d]
	BS9	144.246 ± 19.384[e-i]	2.189 ± 0.402[hi]	783.204 ± 81.283[d-g]	40.193 ± 4.182[h-j]	nd	nd	969.833 ± 32.522[e-g]
	BS10	129.646 ± 11.744[i]	10.146 ± 0.663[cd]	872.127 ± 59.894[c-e]	27.471 ± 1.124j	nd	nd	1039.390 ± 24.388[d-g]
Cheongja 2		187.542 ± 52.411[d-f]	0.090 ± 0.040[i]	437.358 ± 107.879[hi]	72.254 ± 20.033[ef]	3.984 ± 1.460[m]	0.772 ± 0.458[d]	702.000 ± 39.266[h-j]
CV (%)		50.00	99.49	49.46	69.25	84.81	197.82	45.47

C-3-*O*-G: Cyanidin-3-*O*-glucoside; C-3-*O*-Ga: Cyanidin-3-*O*-galactoside; D-3-*O*-G: Delphinidin-3-*O*-glucoside; M-3-*O*-G: Malvidin-3-*O*-glucoside; P-3-*O*-G: Peonidin-3-*O*-glucoside; Pt-3-*O*-G: Petunidin-3-*O*-glucoside; TAC: Total anthocyanin content. nd: Not detected.

Values in the same column marked by different superscript letters are significantly different ($p < 0.05$).

Anthocyanins are presented according to their elusion sequence in the HPLC-DAD chromatogram.

Supplementary Table S3. Isoflavone contents in seeds of black soybeans grown in Korea.

Seed size	Sample Code	Individual isoflavone contents (Mean ± SD, mg/g)												Total contents (Mean ± SD, mg/g)				
		DZG	GLG	GEG	ADZG	AGLG	MDZG	MGLG	DZ	AGLG	GL	MGEG	GE	AGLY	GLY	AcGLY	MaGLY	TIC
Small (N = 6)	BS5	0.141 ± 0.003^{fg}	0.128 ± 0.001^{d}	0.289 ± 0.008^{c-e}	nd	nd	0.611 ± 0.004^{hi}	nd	nd	nd	nd	1.948 ± 0.025^{e-g}	0.123 ± 0.000^{hi}	0.123 ± 0.000^{i}	0.558 ± 0.004^{de}	nd	2.559 ± 0.015^{h-j}	3.241 ± 0.009^{kl}
	BS15	0.068 ± 0.004^{k-m}	0.069 ± 0.003^{j}	0.160 ± 0.007^{k}	nd	nd	0.566 ± 0.025^{j-l}	0.365 ± 0.028^{de}	nd	nd	nd	1.804 ± 0.117^{g-i}	0.124 ± 0.001^{e-i}	0.124 ± 0.000^{i}	0.297 ± 0.002^{lm}	nd	2.735 ± 0.052^{f-h}	3.156 ± 0.041^{k-m}
	BS17	0.125 ± 0.008^{g-i}	nd	0.165 ± 0.006^{jk}	nd	nd	0.534 ± 0.006^{lm}	0.418 ± 0.026^{cd}	nd	0.096 ± 0.003^{b-d}	nd	1.269 ± 0.019^{k}	0.126 ± 0.001^{d-i}	0.126 ± 0.000^{i}	0.291 ± 0.004^{lm}	0.096 ± 0.003^{e-g}	2.221 ± 0.010^{lm}	2.734 ± 0.009^{o}
	BS11	0.323 ± 0.013^{b}	0.195 ± 0.004^{a}	0.289 ± 0.009^{c-e}	0.150 ± 0.006^{a}	nd	1.031 ± 0.017^{a}	nd	0.138 ± 0.004^{a}	0.094 ± 0.002^{b-e}	nd	1.846 ± 0.036^{f-h}	0.136 ± 0.002^{a}	0.274 ± 0.000^{b}	0.808 ± 0.005^{b}	0.244 ± 0.003^{b}	2.877 ± 0.014^{ef}	4.203 ± 0.011^{c-e}
	BS7	0.103 ± 0.004^{h-j}	0.148 ± 0.001^{bc}	0.248 ± 0.005^{f}	nd	nd	0.592 ± 0.002^{ij}	nd	nd	0.096 ± 0.001^{b-d}	nd	1.817 ± 0.010^{g-i}	0.127 ± 0.000^{d-h}	0.127 ± 0.000^{i}	0.499 ± 0.002^{e-g}	0.096 ± 0.000^{e-g}	2.409 ± 0.006^{j-l}	3.130 ± 0.003^{k-m}
	BS21	0.100 ± 0.011^{ij}	0.106 ± 0.009^{e}	0.190 ± 0.010^{ij}	0.116 ± 0.008^{c}	0.113 ± 0.004	0.717 ± 0.020^{de}	nd	0.117 ± 0.004^{ef}	0.091 ± 0.004^{c-f}	0.156 ± 0.004^{a}	2.009 ± 0.073^{d-f}	0.124 ± 0.001^{e-i}	0.397 ± 0.002^{a}	0.395 ± 0.001^{i-k}	0.321 ± 0.002^{a}	2.726 ± 0.038^{f-h}	3.839 ± 0.020^{f-h}
Medium (N = 6)	BS14	0.061 ± 0.003^{k-m}	0.084 ± 0.004^{gh}	0.219 ± 0.003^{gh}	nd	nd	0.478 ± 0.005^{o}	nd	nd	0.096 ± 0.001^{bc}	nd	1.654 ± 0.033i	0.128 ± 0.001^{c-e}	0.128 ± 0.000^{i}	0.364 ± 0.000^{jk}	0.096 ± 0.000^{e-g}	2.132 ± 0.020^{mn}	2.720 ± 0.012^{o}
	BS18	0.046 ± 0.012^{mn}	0.151 ± 0.007^{b}	0.275 ± 0.009^{d-f}	nd	nd	0.483 ± 0.007^{no}	nd	nd	nd	nd	1.914 ± 0.047^{e-g}	0.128 ± 0.001^{c-f}	0.128 ± 0.000^{i}	0.472 ± 0.002^{f-h}	nd	2.397 ± 0.028^{j-l}	2.997 ± 0.017^{l-n}
	BS13	0.022 ± 0.005^{n}	0.075 ± 0.002^{h-j}	0.152 ± 0.005^{k}	nd	nd	0.455 ± 0.009^{o}	nd	nd	nd	nd	1.436 ± 0.045^{j}	0.130 ± 0.000^{b-d}	0.130 ± 0.000^{i}	0.248 ± 0.00^{1m}	nd	1.891 ± 0.026^{n}	2.269 ± 0.017^{p}
	BS23	0.054 ± 0.004^{lm}	0.078 ± 0.001^{h-j}	0.204 ± 0.008^{hi}	nd	nd	0.522 ± 0.009^{mn}	nd	nd	0.091 ± 0.004^{d-f}	nd	1.893 ± 0.058^{e-g}	0.123 ± 0.000^{i}	0.123 ± 0.000^{i}	0.336 ± 0.004^{kl}	0.091 ± 0.000^{f-h}	2.415 ± 0.034^{i-l}	2.965 ± 0.02^{1-o}
	BS22	0.055 ± 0.005^{lm}	0.080 ± 0.001^{h-j}	0.201 ± 0.007^{hi}	nd	nd	0.534 ± 0.008^{lm}	nd	0.115 ± 0.004^{ef}	0.092 ± 0.003^{c-f}	0.154 ± 0.001^{a}	1.937 ± 0.046^{e-g}	0.124 ± 0.001^{f-i}	0.393 ± 0.002^{a}	0.336 ± 0.003^{kl}	0.092 ± 0.000^{e-h}	2.471 ± 0.027^{i-k}	3.292 ± 0.014^{jk}
	BS2	0.088 ± 0.006^{jk}	0.132 ± 0.003^{d}	0.206 ± 0.003^{hi}	nd	nd	0.450 ± 0.004^{o}	nd	0.117 ± 0.001^{ef}	nd	nd	0.993 ± 0.014^{l}	0.123 ± 0.000^{hi}	0.240 ± 0.001^{gh}	0.426 ± 0.002^{h-j}	nd	1.443 ± 0.007^{o}	2.110 ± 0.005^{p}
Large (N = 12)	BS20	0.294 ± 0.008^{bc}	0.0136 ± 0.001^{cd}	0.252 ± 0.100^{f}	nd	nd	1.021 ± 0.020^{a}	nd	0.123 ± 0.002^{cd}	0.099 ± 0.003^{b}	nd	2.287 ± 0.042^{bc}	0.128 ± 0.001^{c-g}	0.251 ± 0.001^{ef}	0.682 ± 0.004^{c}	0.099 ± 0.000^{ef}	3.308 ± 0.015^{b}	4.340 ± 0.014^{c}
	BS1	0.353 ± 0.030^{a}	0.102 ± 0.005^{ef}	0.487 ± 0.027^{b}	0.128 ± 0.003^{b}	nd	0.798 ± 0.023^{b}	nd	0.118 ± 0.001^{ef}	0.093 ± 0.000^{c-e}	nd	2.413 ± 0.080^{b}	0.127 ± 0.001^{d-h}	0.245 ± 0.000^{f-h}	0.941 ± 0.004^{a}	0.222 ± 0.002^{c}	3.211 ± 0.041^{bc}	4.618 ± 0.026^{b}
	BS6	0.265 ± 0.001^{cd}	0.104 ± 0.001^{e}	0.522 ± 0.003^{a}	nd	nd	0.765 ± 0.018^{bc}	0.639 ± 0.030^{a}	nd	0.108 ± 0.002^{a}	nd	3.245 ± 0.108^{a}	0.129 ± 0.001^{cd}	0.129 ± 0.000^{i}	0.891 ± 0.001^{a}	0.108 ± 0.000^{d}	4.649 ± 0.049^{a}	5.777 ± 0.037^{a}
	BS4	0.197 ± 0.006^{e}	0.092 ± 0.001^{fg}	0.247 ± 0..007^{fg}	nd	nd	0.685 ± 0.008^{ef}	nd	nd	nd	nd	1.687 ± 0.036^{hi}	0.127 ± 0.001^{d-i}	0.127 ± 0.000^{i}	0.537 ± 0.003^{de}	nd	2.372 ± 0.020^{j-l}	3.035 ± 0.013^{k-n}
	BS24	0.249 ±	0.075 ±	0.271 ±	nd	nd	0.760 ±	0.223 ±	0.113 ±	0.094 ±	nd	2.026 ±	0.124 ±	0.237 ±	0.595 ±	0.094 ±	3.009 ±	3.936 ±

		0.008^{d}	0.002^{h-j}	0.005^{ef}			0.006^{bc}	0.039^{f}	0.003^{f}	0.002^{b-e}		0.024^{de}	0.003^{g-i}	0.000^{h}	0.003^{d}	0.000^{e-h}	0.016^{c-e}	0.013^{fg}
	BS19	0.128 ± 0.008^{g-i}	0.079 ± 0.004^{h-j}	0.209 ± 0.007^{hi}	nd	nd	0.753 ± 0.008^{cd}	0.452 ± 0.053^{bc}	0.120 ± 0.001^{de}	0.096 ± 0.001^{b-d}	nd	1.985 ± 0.036^{d-f}	0.127 ± 0.000^{d-h}	0.246 ± 0.000^{fg}	0.417 ± 0.002^{h-j}	0.096 ± 0.000^{e-g}	3.190 ± 0.023^{b-d}	3.949 ± 0.019^{e-g}
	BS12	0.043 ± 0.005^{mn}	0.074 ± 0.004^{h-j}	0.143 ± 0.003^{k}	nd	nd	0.543 ± 0.002^{k-m}	0.308 ± 0.001^{e}	0.119 ± 0.001^{de}	nd	nd	1.468 ± 0.006^{j}	0.126 ± 0.001^{d-i}	0.245 ± 0.000^{fg}	0.260 ± 0.001^{m}	nd	2.319 ± 0.002^{k-m}	2.825 ± 0.002^{no}
	BS8	0.118 ± 0.003^{g-i}	0.097 ± 0.003^{ef}	0.308 ± 0.005^{c}	nd	nd	0.578 ± 0.003^{i-k}	0.409 ± 0.375^{cd}	0.119 ± 0.001^{de}	0.090 ± 0.000^{ef}	nd	2.218 ± 0.010^{c}	0.133 ± 0.000^{ab}	0.251 ± 0.001^{d-f}	0.523 ± 0.001^{ef}	0.090 ± 0.000^{gh}	3.205 ± 0.014^{bc}	4.070 ± 0.009^{d-f}
	BS16	0.134 ± 0.011^{gh}	0.072 ± 0.001^{ij}	0.303 ± 0.012^{cd}	nd	nd	0.558 ± 0.015^{j-m}	0.375 ± 0.015^{d}	nd	nd	nd	2.052 ± 0.078^{de}	0.129 ± 0.000^{b-d}	0.129 ± 0.000^{i}	0.505 ± 0.006^{e-g}	nd	2.985 ± 0.037^{de}	3.618 ± 0.027^{hi}
	BS3	0.078 ± 0.001^{j-l}	0.081 ± 0.003^{g-i}	0.253 ± 0.003^{f}	nd	nd	0.525 ± 0.006^{m}	nd	nd	nd	nd	1.904 ± 0.037^{e-g}	0.129 ± 0.004^{b-d}	0.129 ± 0.000^{i}	0.411 ± 0.001^{h-j}	nd	2.428 ± 0.022^{i-l}	2.968 ± 0.014^{m-o}
	BS9	0.119 ± 0.009^{g-i}	0.068 ± 0.004^{j}	0.262 ± 0.007^{ef}	nd	nd	0.632 ± 0.012^{gh}	0.486 ± 0.039^{b}	0.127 ± 0.001^{bc}	0.099 ± 0.002^{b}	nd	2.289 ± 0.045^{bc}	0.130 ± 0.001^{b-d}	0.257 ± 0.000^{c-e}	0.450 ± 0.002^{g-i}	0.099 ± 0.003^{e}	3.407 ± 0.018^{b}	4.212 ± 0.017^{cd}
	BS10	0.176 ± 0.10^{e}	0.084 ± 0.002^{gh}	0.264 ± 0.011^{ef}	nd	nd	0.655 ± 0.015^{fg}	0.189 ± 0.001^{f}	0.131 ± 0.001^{b}	0.087 ± 0.000^{f}	nd	1.787 ± 0.041^{g-i}	0.133 ± 0.000^{ab}	0.264 ± 0.001^{c}	0.524 ± 0.005^{ef}	0.087 ± 0.003^{h}	2.631 ± 0.020^{g-i}	3.506 ± 0.013^{ij}
Cheongja 2		0.170 ± 0.008^{ef}	0.097 ± 0.003^{ef}	0.285 ± 0.010^{c-e}	nd	nd	0.667 ± 0.008^{fg}	nd	0.128 ± 0.001^{bc}	0.096 ± 0.001^{bc}	nd	2.126 ± 0.036^{cd}	0.131 ± 0.001^{bc}	0.259 ± 0.000^{cd}	0.552 ± 0.003^{de}	0.096 ± 0.003^{e-g}	2.793 ± 0.020^{e-g}	3.701 ± 0.012^{g-i}
CV (%)		65.811	38.986	34.743	12.944	-	24.295	33.640	5.933	5.068	0.818	22.133	2.590	41.496	37.176	55.193	22.693	23.060

AcGLY: Total acetylglycoside content; AGLY: Total aglycone content ADZG: Acetyldaidzin; AGEG: Acetylgenistin; AGLG: Acetylglycitin; DZ: Daidzein; DZG: Daidzin; GE: Genistein; GEG: Genistin; GL: Glycitein; GLY: Total *β*-glycoside content; GLG: Glycitin; MaGLY: Total malonylglycoside content; MDZG: Malonyldaidzin; MGEG: Malonylgenistin; MGLG: Malonylglycitin; TIC: Total isoflavone content.

nd: Not detected.

Isoflavones are presented according to their elusion sequence in the HPLC-DAD chromatogram.

Values in the same column marked by different superscript letters are significantly different ($p < 0.05$).

Supplementary Table S4. Pearson correlation coefficient (r) value (lower bound) for the pair-wise correlation between anthocyanins, isoflavones, total phenolic content and antioxidant activities in 25 black soybean varieties. The corresponding *p*-values are presented in the upper bound of the table.

Variables	D-3-*O*-G	C-3-*O*-Ga	C-3-*O*-G	Pt-3-*O*-G	P-3-*O*-G	M-3-*O*-G	TAC	DZG	GLG	GEG	ADZG	AGLG	MDZG	MGLG	DZ	AGEG	GL	MGEG	GE	TIC	TPC	FRAP	DPPH	ABTS
D-3-*O*-G	1	0.043	0.006	0.017	0.009	0.369	0.000	0.856	0.710	0.622	0.176	0.995	0.629	0.441	0.387	0.498	0.806	0.196	0.959	0.585	0.493	0.040	0.756	0.024
C-3-*O*-Ga	0.408	1	< 0.0001	0.089	0.002	0.263	< 0.0001	0.658	0.566	0.795	0.070	0.325	0.763	0.446	0.782	0.574	0.975	0.265	0.965	0.506	0.632	0.265	0.821	0.204
C-3-*O*-G	0.537	0.826	1	0.078	0.000	0.317	< 0.0001	0.912	0.435	0.959	0.071	0.492	0.532	0.240	0.711	0.481	0.808	0.116	0.665	0.283	0.986	0.070	0.501	0.076
Pt-3-*O*-G	0.471	0.347	0.359	1	< 0.0001	< 0.0001	0.006	0.840	0.739	0.870	0.461	0.880	0.904	0.154	0.265	0.679	0.869	0.677	0.706	0.794	0.190	0.105	0.057	0.278
P-3-*O*-G	0.508	0.596	0.671	0.792	1	0.000	< 0.0001	0.717	0.916	0.605	0.245	0.736	0.828	0.319	0.081	0.916	0.828	0.614	0.270	0.817	0.366	0.021	0.111	0.208
M-3-*O*-G	0.188	0.233	0.209	0.883	0.672	1	0.085	0.400	0.384	0.579	0.456	0.624	0.614	0.137	0.152	0.978	0.477	0.758	0.788	0.334	0.209	0.434	0.215	0.822
TAC	0.688	0.807	0.970	0.537	0.771	0.351	1	0.906	0.623	0.998	0.063	0.548	0.552	0.571	0.508	0.458	0.785	0.119	0.797	0.370	0.919	0.034	0.376	0.044
DZG	0.038	-0.093	-0.023	-0.043	-0.076	-0.176	-0.025	1	0.095	0.000	0.009	0.662	< 0.0001	0.838	0.069	0.031	0.327	0.008	0.145	< 0.0001	0.430	0.899	0.701	0.868
GLG	0.078	-0.120	-0.163	0.070	0.022	0.182	-0.103	0.341	1	0.156	0.036	0.802	0.027	0.017	0.365	0.959	0.896	0.455	0.234	0.337	0.152	0.163	0.153	0.570
GEG	0.104	-0.055	-0.011	-0.034	-0.109	-0.116	-0.001	0.693	0.292	1	0.165	0.461	0.039	0.348	0.845	0.147	0.324	< 0.0001	0.198	< 0.0001	0.266	0.947	0.167	0.789
ADZG	-0.280	-0.369	-0.367	-0.154	-0.241	-0.156	-0.377	0.513	0.422	0.286	1	0.016	0.005	0.180	0.067	0.213	0.155	0.507	0.270	0.091	0.368	0.670	0.497	0.950
AGLG	-0.001	-0.205	-0.144	-0.032	-0.071	-0.103	-0.126	-0.092	0.053	-0.154	0.477	1	0.606	0.462	0.391	0.515	0.000	0.837	0.337	0.666	0.266	0.283	0.397	0.907
MDZG	0.102	0.064	0.131	0.025	0.046	-0.106	0.125	0.869	0.441	0.416	0.548	0.108	1	0.938	0.014	0.014	0.923	0.012	0.122	< 0.0001	0.695	0.200	0.843	0.335
MGLG	-0.161	0.160	0.244	-0.294	-0.208	-0.306	0.119	0.043	-0.475	0.196	-0.277	-0.154	0.017	1	0.927	0.410	0.285	0.076	0.516	0.028	0.992	0.498	0.471	0.437
DZ	-0.181	0.058	-0.078	-0.232	-0.356	-0.295	-0.139	0.370	0.189	0.041	0.372	0.179	0.485	0.019	1	0.030	0.221	0.688	0.237	0.095	0.560	0.237	0.999	0.187
AGEG	0.142	0.118	0.148	0.087	0.022	-0.006	0.155	0.432	-0.011	0.299	0.258	0.137	0.485	0.172	0.434	1	0.339	0.023	0.311	0.002	0.699	0.708	0.256	0.319
GL	-0.052	0.006	-0.051	-0.035	0.046	-0.149	-0.057	-0.204	-0.027	-0.206	0.293	0.697	-0.020	-0.223	0.254	0.199	1	0.859	0.137	0.888	0.072	0.082	0.320	0.826
MGEG	0.268	0.232	0.323	0.088	0.106	-0.065	0.320	0.518	0.156	0.789	0.139	0.043	0.495	0.362	0.084	0.453	0.037	1	0.327	< 0.0001	0.876	0.224	0.827	0.043
GE	-0.011	0.009	0.091	-0.079	-0.230	-0.057	0.054	0.300	0.247	0.266	0.230	-0.200	0.318	0.136	0.246	0.211	-0.306	0.205	1	0.137	0.021	0.591	0.558	0.100
TIC	0.115	0.140	0.224	-0.055	-0.049	-0.202	0.187	0.729	0.200	0.773	0.346	0.091	0.723	0.439	0.341	0.581	0.030	0.916	0.306	1	0.894	0.394	0.850	0.151
TPC	0.144	-0.101	-0.004	-0.271	-0.189	-0.260	-0.021	0.165	0.295	0.231	0.188	-0.231	0.082	-0.002	-0.122	-0.081	-0.366	-0.033	0.461	0.028	1	0.973	0.000	0.433
FRAP	0.413	0.232	0.368	0.332	0.459	0.164	0.426	0.027	0.288	-0.014	0.090	0.224	0.265	-0.142	-0.245	0.079	0.354	0.252	0.113	0.178	0.007	1	0.445	0.004
DPPH	-0.065	-0.048	-0.141	-0.385	-0.327	-0.257	-0.185	0.081	0.294	0.285	0.143	-0.177	-0.042	0.151	0.000	-0.236	-0.207	-0.046	0.123	0.040	0.673	-0.160	1	0.259
ABTS	0.450	0.263	0.361	0.226	0.261	0.047	0.407	0.035	-0.119	0.056	-0.013	0.025	0.201	0.163	-0.273	0.208	0.046	0.407	0.336	0.296	0.164	0.551	-0.235	1

ABTS: ABTS-radical scavenging activity; ADZG: Acetyldaidzin; AGEG: Acetylgenistin; AGLG: Acetylglycitin; C-3-*O*-G: Cyanidin-3-*O*-glucoside; C-3-*O*-Ga: Cyanidin-3-*O*-galactoside; DPPH: DPPH-radical scavenging activity; DZ: Daidzein; DZG: Daidzin; D-3-*O*-G: Delphinidin-3-*O*-glucoside; FRAP: Ferric reducing antioxidant power; GE: Genistein; GEG: Genistin; GL: Glycitein; GLG: Glycitin; MDZG: Malonyldaidzin; MGEG: Malonylgenistin; MGLG: Malonylglycitin; M-3-*O*-G: Malvidin-3-*O*-glucoside; P-3-*O*-G: Peonidin-3-*O*-glucoside; Pt-3-*O*-G: Petunidin-3-*O*-glucoside; TAC: Total anthocyanin content; TIC: Total isoflavone content; TPC, Total phenolic content.
